# Supplementary material for: Carbon footprint of global natural gas supplies to China
Source: Nat Commun. 2020 Feb 11;11:824. doi: 10.1038/s41467-020-14606-4 (PMC7012848; doi:10.1038/s41467-020-14606-4)
Supplement: Supplementary file 2 — Supplementary Information [file 41467_2020_14606_MOESM2_ESM.pdf]

Supplementary Information

Carbon footprint of global natural gas supplies to China

Gan et al.

Supplementary Figures

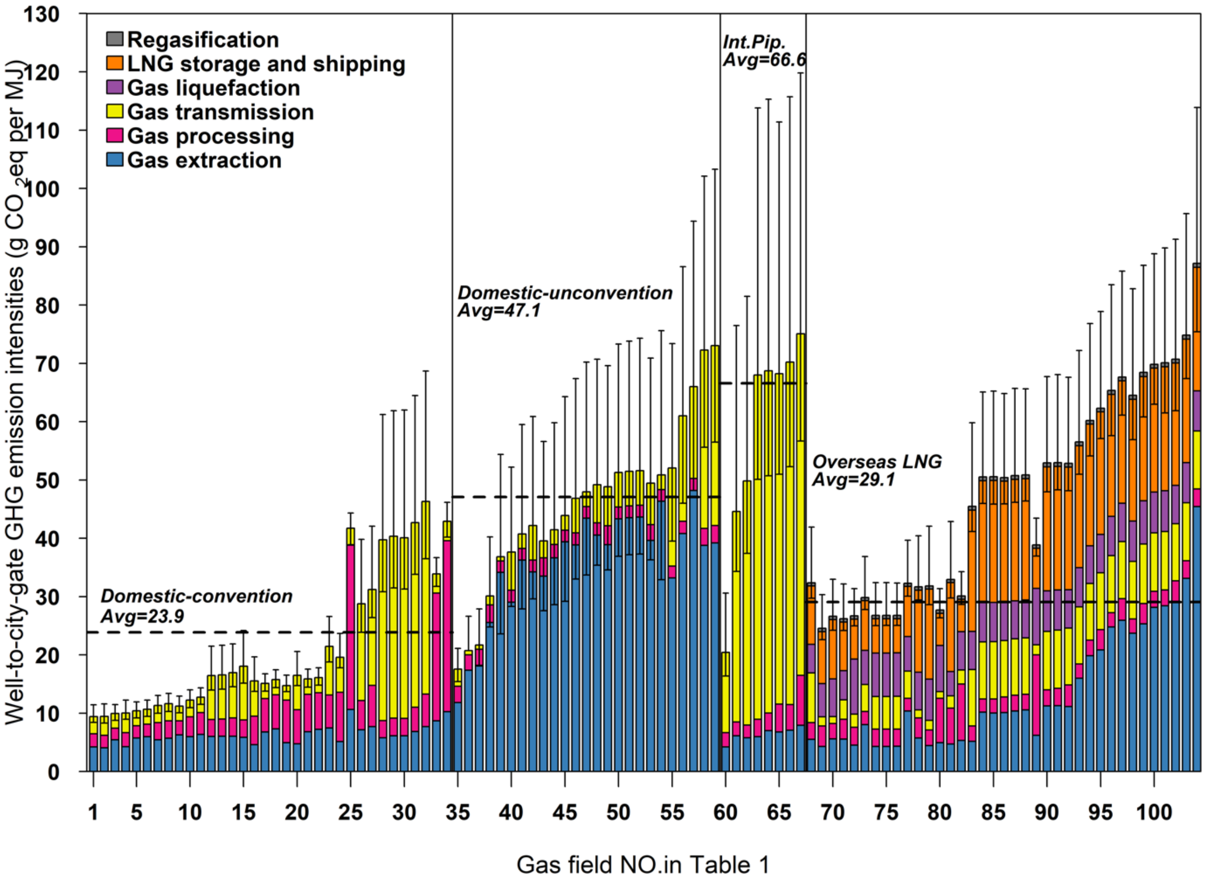

Supplementary Fig. 1 Well-to-city-gate GHG emission intensities of individual natural gas suppliers for China using 20-year timeframe global warming potential (GWP_{20}). Different colors show the breakdown of emissions by individual processes. Error bars show the 90% confidence interval of results from the Monte Carlo Simulations, see Supplementary Discussion 2 Uncertainty Analysis for details.

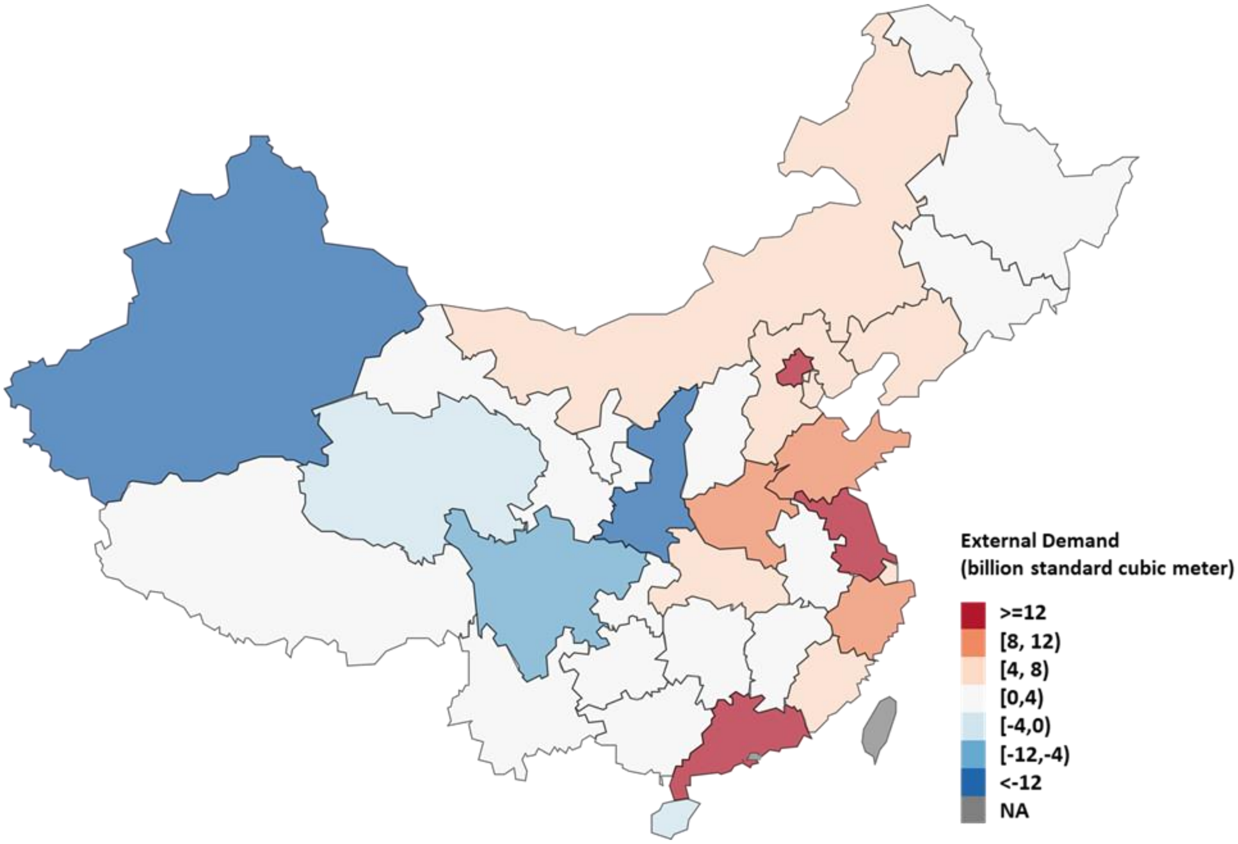

Supplementary Fig. 2 External demand of natural gas of different provinces in China for 2016. The external demand is calculated as consumption minus domestic production of the province[1], indicating the total amount of natural gas imports from other provinces or other countries in 2016. Provinces in red are regions that require gas imports, while provinces in blue with negative values of external demand, are regions that export natural gas to other provinces.

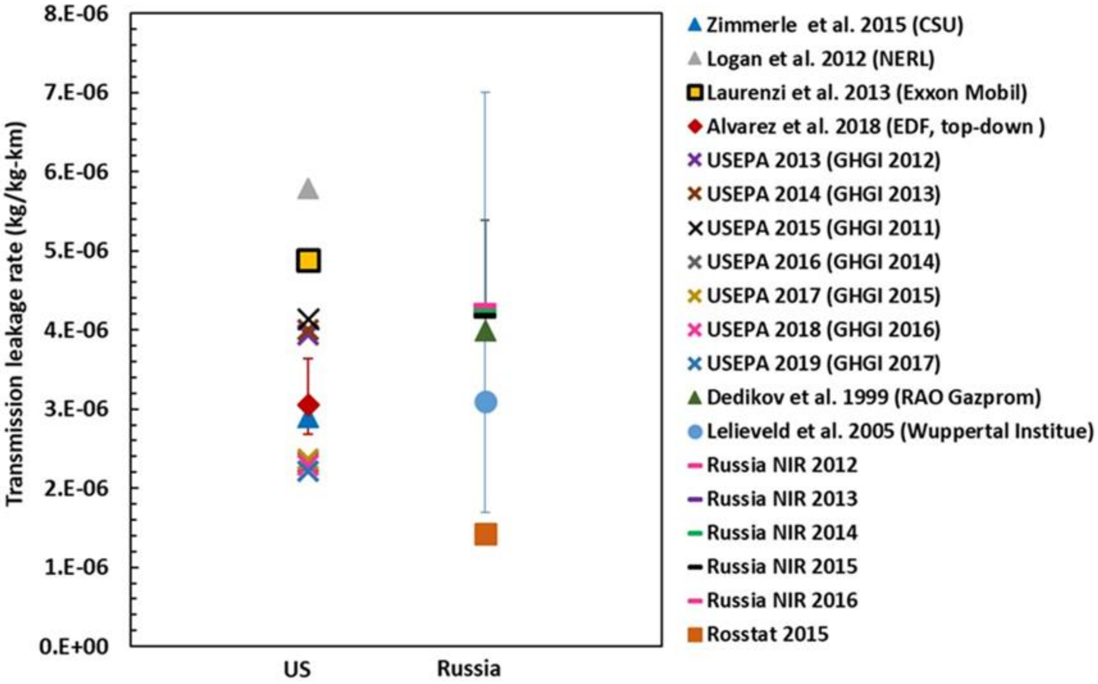

Supplementary Fig. 3 Literature estimates of leakage rates of the U.S. and Russia pipeline system.
Data sources: Zimmerle et al. 2015[2], Logan et al. 2012[3], Laurenzi et al. 2013[4], Alvarez et al. 2018[5], USEPA 2013[6], USEPA 2014[7], USEPA 2015[8], USEPA 2016[9], USEPA 2017[10], USEPA 2018[11], USEPA 2019[12], Dedikov et al. 1999[13], Lelieveld et al.[14,15], Russia NIR 2012[16], Russia NIR 2013[17], Russia NIR 2014[18], Russia NIR 2015[19], Russia NIR 2016[20], Rosstat 2015[21].

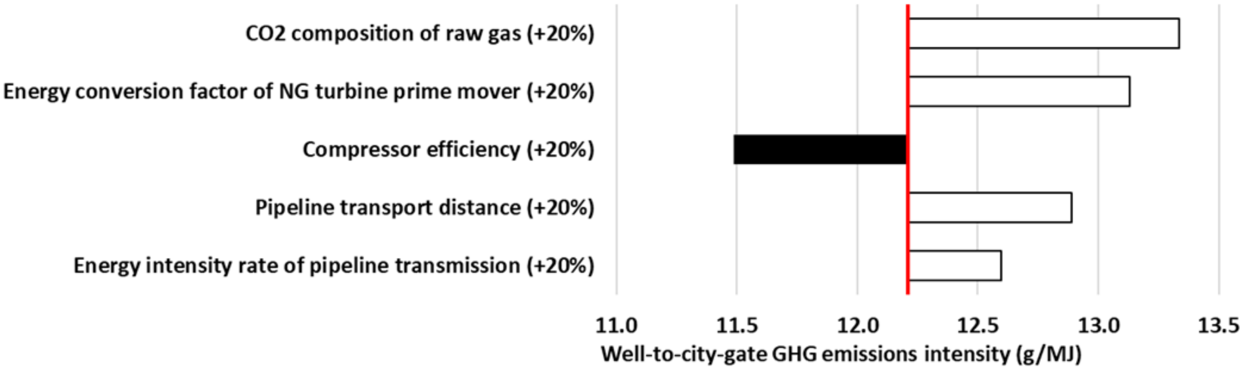

Supplementary Fig. 4 Variation of GHG emissions results responding to the uncertain input of top 5 sensitive parameters for a domestic conventional gas field, Ya.

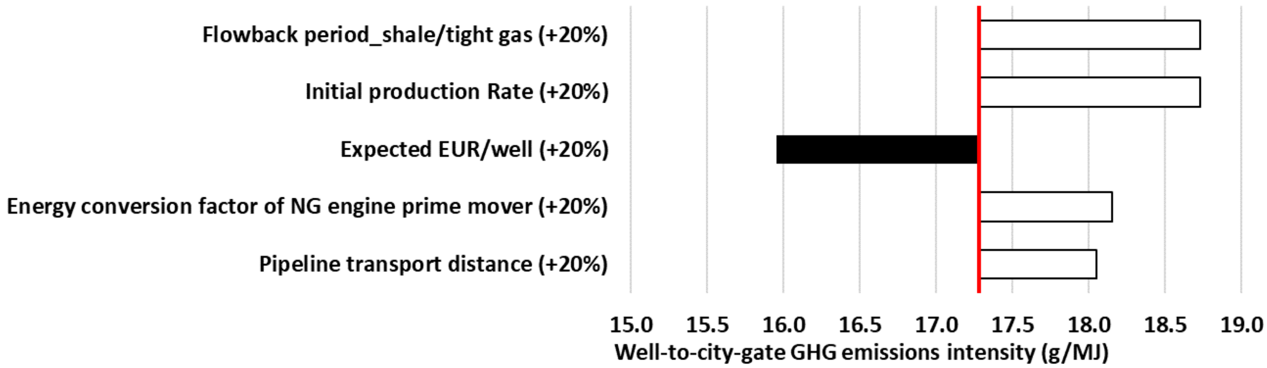

Supplementary Fig. 5 Variation of GHG emissions results responding to the uncertain input of top 5 sensitive parameters for a domestic unconventional gas field, Sulige.

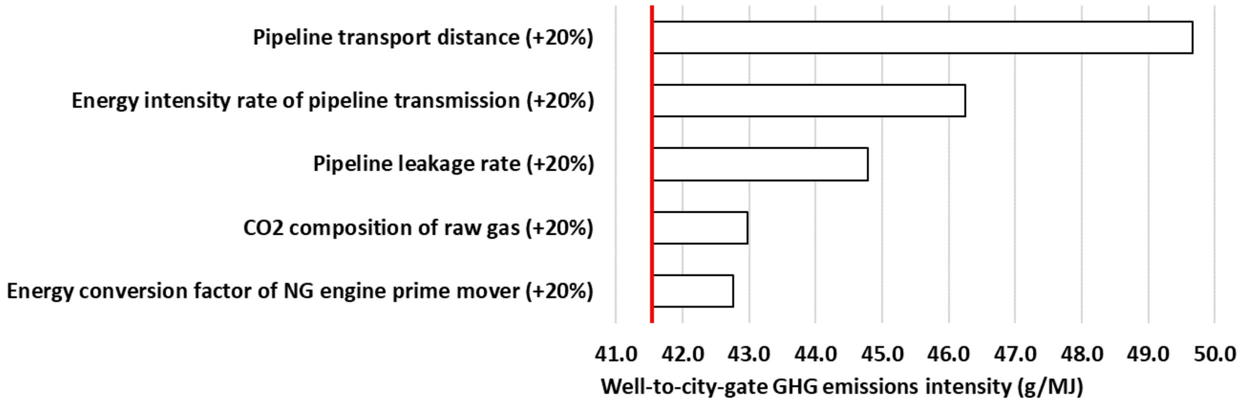

Supplementary Fig. 6 Variation of GHG emissions results responding to the uncertain input of top 5 sensitive parameters for an international pipeline gas field, Galkynysh.

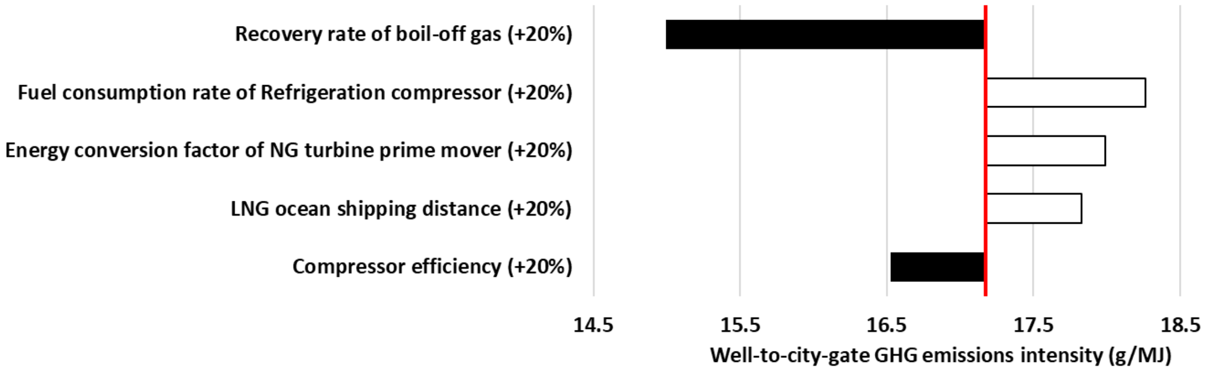

Supplementary Fig. 7 Variation of GHG emissions results responding to the uncertain input of top 5 sensitive parameters for an LNG source, Qatargas North field.

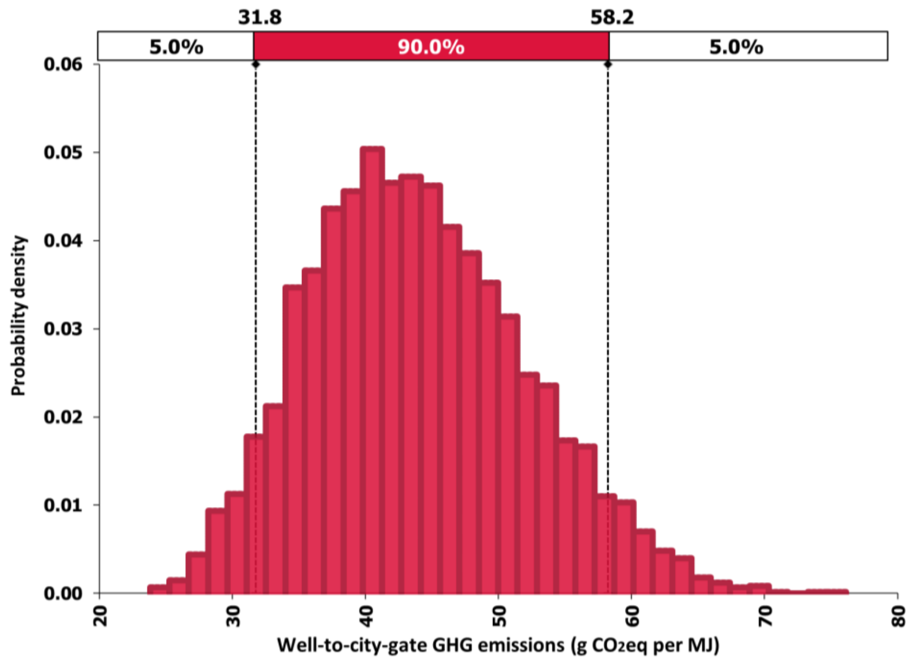

Supplementary Fig. 8 Probability density function (PDF) of well-to-city-gate GHG emissions in GWP_{100} for gas supply from Galkynysh gas field. The PDF is generated based on results of 5000 times of Monte Carlo simulations. GHG emissions in the figure are characterized with 100 years global warming potential (GWP_{100}) factors.

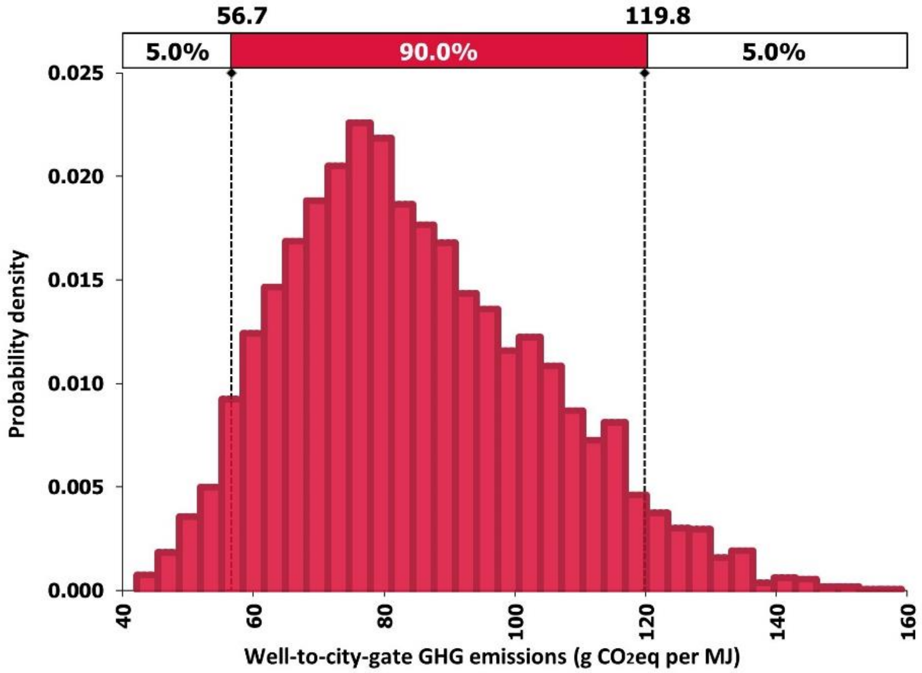

Supplementary Fig. 9 Probability density function (PDF) of well-to-city-gate GHG emissions in GWP_{20} for gas supply from Galkynysh gas field. The PDF is generated based on results of 5000 times of Monte Carlo simulations. GHG emissions in the figure are characterized with 20 years global warming potential (GWP_{20}) factors.

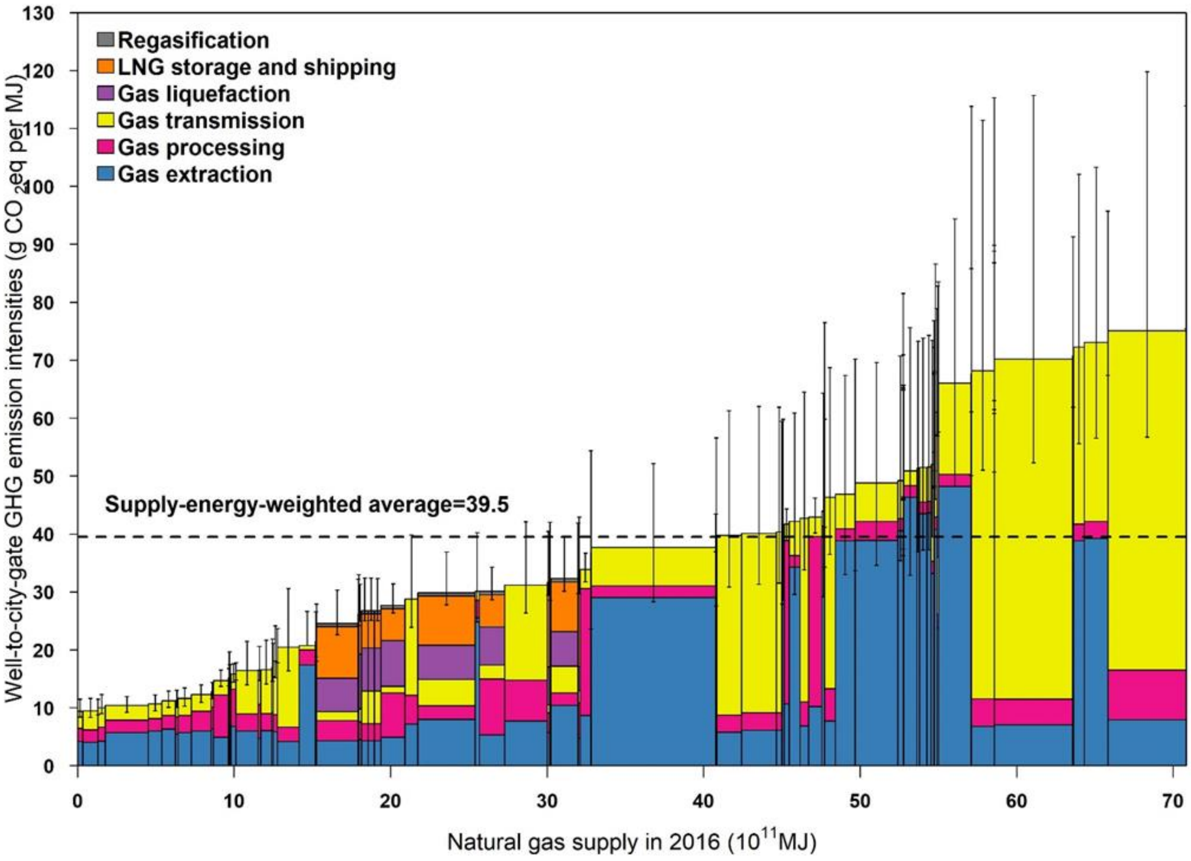

Supplementary Fig. 10 Well-to-city-gate GHG emission intensity supply curve of natural gas for China of 2016 in GWP_{20}. Error bars show the 90% confidence interval of results from the Monte Carlo Simulations, see Supplementary Discussion 2 Uncertainty Analysis for details.

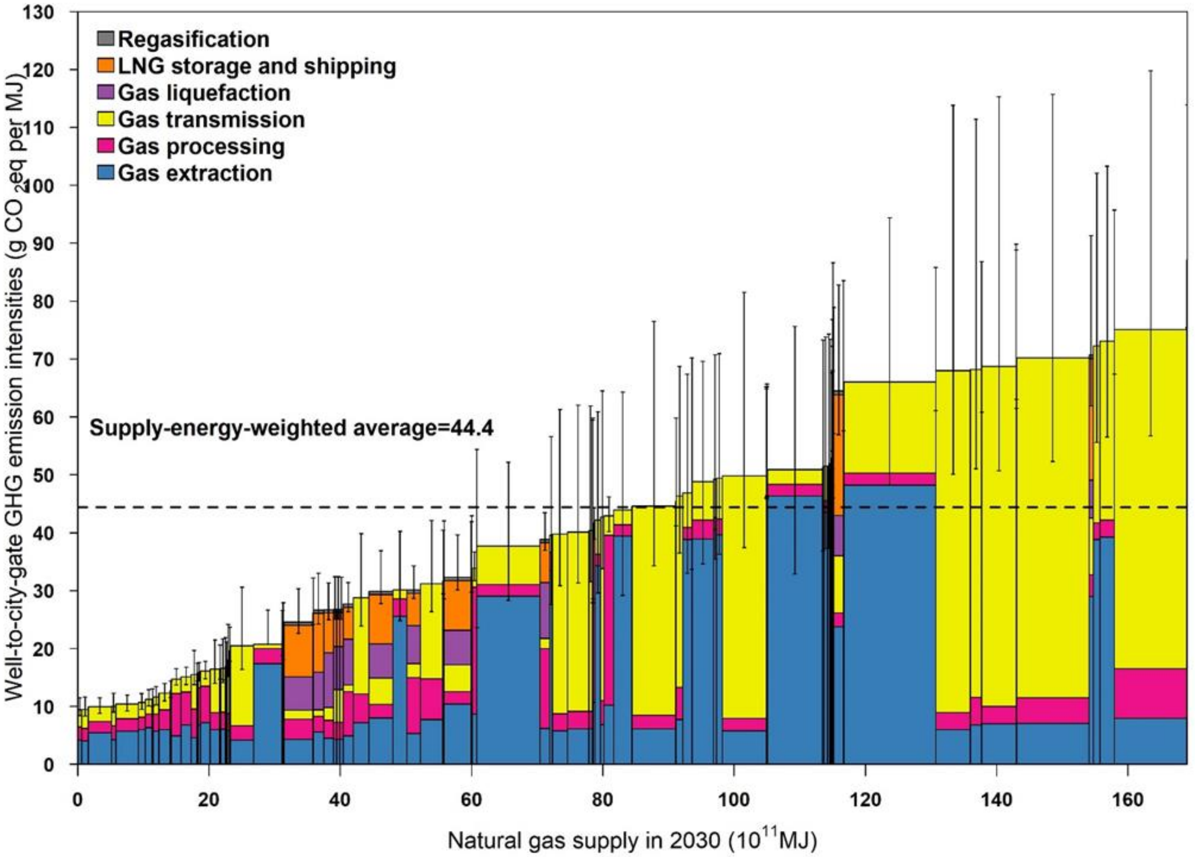

Supplementary Fig. 11 Well-to-city-gate GHG emission intensity supply curve of natural gas for China of 2030 in GWP_{20}. Error bars show the 90% confidence interval of results from the Monte Carlo Simulations, see Supplementary Discussion 2 Uncertainty Analysis for details.

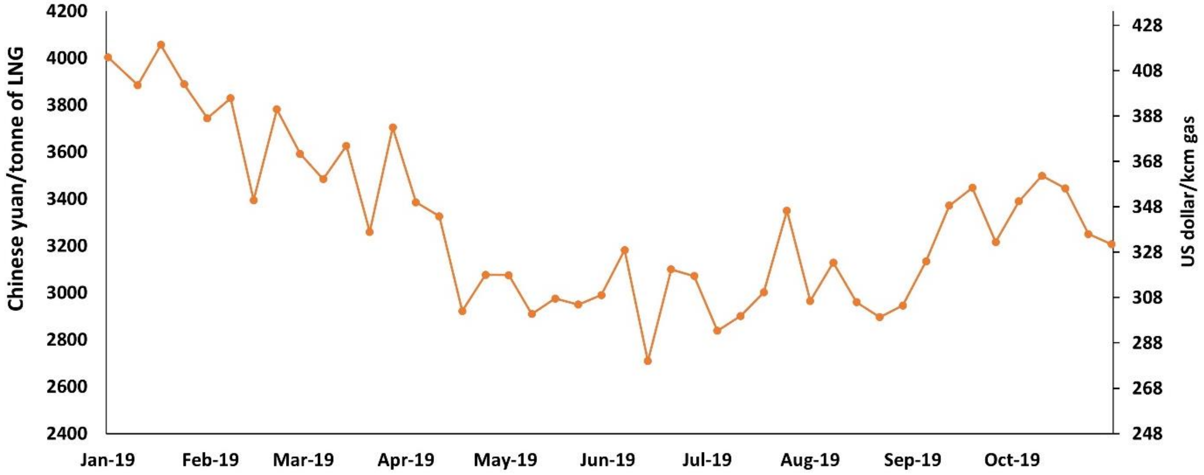

Supplementary Fig. 12 Delivered ex-ship (DES) price of LNG imports for China in 2019.
Data source: Shanghai Petroleum and Gas Exchange (SHPGX)[22]

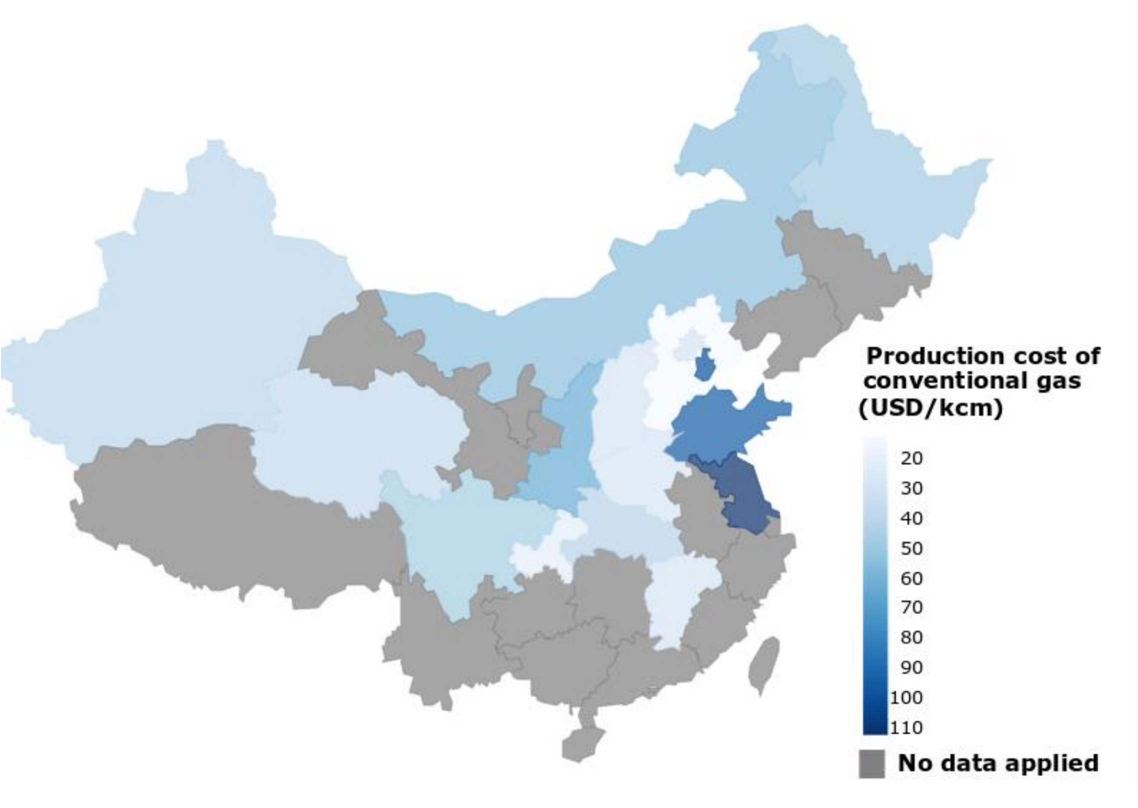

Supplementary Fig. 13 Production cost of conventional gas in different provinces of China in 2015.
Data sources: Rioux et al[102], Zhang et al[23].

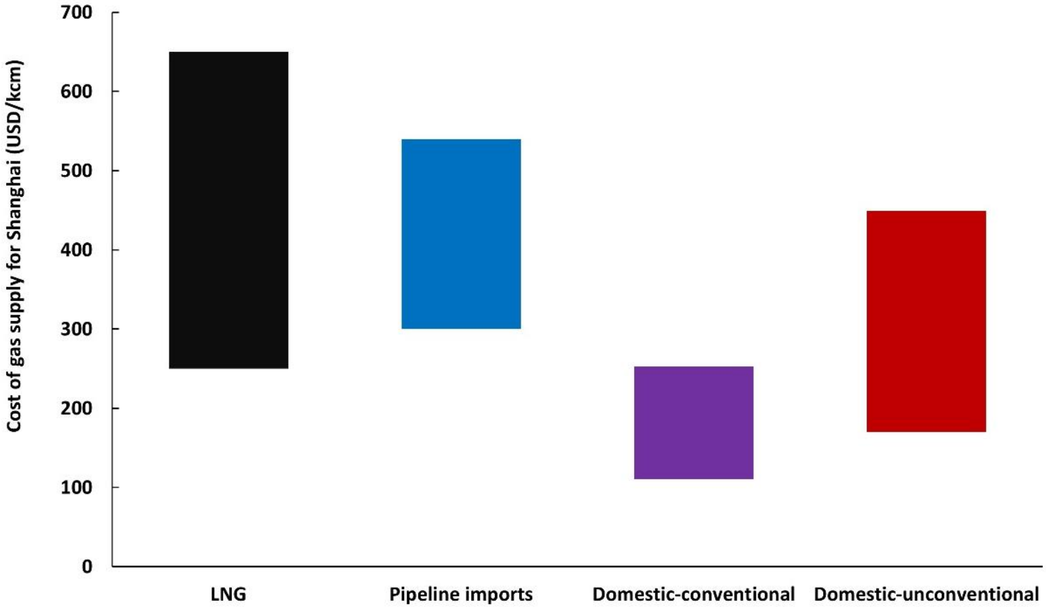

Supplementary Fig. 14 Estimated range of cost for different categories of gas supply to Shanghai
Data sources: Rioux et al[102], Zhang et al[23], International Trade Centre (ITC)[105], General Administration of Customs of China[106], Shanghai Petroleum and Gas Exchange (SHPGX)[22], General Electric(GE)[24].

Supplementary Tables

Supplementary Table 1 Comparison of characteristics of natural gas pipeline system in different countries

	**Year of Pipeline installed**	**Operating pressure**	**Maintenance and mitigation practice**
China's pipeline system	2000s	6.3~12 MPa	No regulation and measurements on methane emissions
U.S pipeline system	Median at 1960s	3.5~10 MPa	Well-maintained with deployment of emissions mitigation practices
Russian gas export pipeline system	Median at 1980s	7.5 Mpa	Monitored leaks, promoted and deployed emissions mitigation practices

Data sources: ANL 2007[37], INGAA[38], Lelieveld et al[14,15], Balcombe et al[39].

Supplementary Table 2 Probability distributions of parameters used in Monte Carlo simulation (field-specific parameters excluded)

Parameter	Uncertainty range	Data sources
Flaring rate_conventional gas (%)	Triangular(41,51,61)[a]	NETL[25,26]
Flaring rate_unconventional gas (%)	Triangular(12,15,18)	NETL[25,26]
Flowback period_shale/tight gas (days)	Triangular(3,7,10)	NETL[25,26]
Compressor efficiency (%)	Triangular(70,75,85)	Simpson[27]
Energy conversion factor of NG engine prime mover	Triangular(2.38,3.11,3.27)	OPGEE[28]
Energy conversion factor of NG turbine prime mover	Triangular(2.76,3.62,4.08)	OPGEE[28]
Flaring efficiency (%)	Triangular(80, 98,100)	J Willis et al[29]
Energy intensity rate of pipeline transmission (MJ/tonne-km)	Triangular(0.29,1.19,1.62)	Müller-Syring[30], NETL[25,26], GREET
Pipeline leakage rate (kg/kg-km)	Uniform(2.2E-06, 5.84E-06) for U.S. pipeline system Triangular(1.4E-06, 3.0E-06, 7.1E-06) for others	Zimmerle et al. 2015[2], Logan et al. 2012[3], Laurenzi et al. 2013[4], Alvarez et al. 2018[5], USEPA 2013~2019[6-12], Dedikov et al. 1999[13], Lelieveld et al.[14,15], Russia NIR 2012~2016[16-20], Rosstat 2015[21]
LNG boil-off rate (%)	Triangular(0.08,0.10,0.15)	Dobrota et al[31], Głomski and Michalski[32], Sedlaczek[33]
Recovery rate of boil-off gas (%)	Triangular(70,80,90)	Kwak et al[34]
Ocean tanker average speed (km/hour)	Triangular(8.8,12.5,14.1)	IMO[35,36]

a Triangular(a,b,c) means the value input in the model is a triangular distribution, with the lower bound of a, upper bound of c, and most likely value of b.

Supplementary Table 3 Import price of intentional pipeline gas in 2018 and estimates of transmission costs

Country	Import price 2018 (USD/kcm)	Cost of gas transmission within China (USD/kcm)							
		To Guangdong	To Fujian	To Shanghai	To Zhejiang	To Guangxi	To Henan	To Yunnan	Mean
Turkmenistan	393	134	140	138	137	148	103	--	128
Uzbekistan	374								
Kazakhstan	358								
Myanmar	603	--	--	--	--	46	--	16	30

Data sources: International Trade Centre (ITC)[105], General Administration of Customs of China[106], Rioux et al[102], Zhang et al[23].

Supplementary Table 4 Key assumptions for calculating extraction-associated emissions

Parameters	Unit	Value	Data sources
Well depth	meter	Field-specific input [a]	
EUR	billion cubic meter	Field-specific input	
Initial production rate	thousand cubic meter/day	Field-specific input	
Average production rate	thousand cubic meter/day	Field-specific input	
Average Penetration Rate	meter per hour	15.2 (14.6~16.8)	Jiang et al[40]
Drilling Rig Capacity	kilowatts	3390 (1860~4920)	Jiang et al[40]
Flaring rate_conventional gas	%	51	NETL[25,26]
Flaring rate_unconventional gas	%	15	NETL[25,26]
Flaring efficiency	%	98	IPCC[41]
Well completion_conventional gas	thousand cubic meter per episode	1.05	NETL[25,26]
Well completion_CBM	thousand cubic meter per episode	1.40	NETL[25,26]
Flowback period_shale&tight gas	day	7 (3~10)	NETL[25,26]
Liquid Uploading per episode	thousand cubic meter	1.02	NETL[25,26]
Times of uploading of well life	times	930	NETL[25,26]
Well workover_conventional gas	cubic meter per episode	69	NETL[25,26]
Well workover_CBM	thousand cubic meter per episode	1.40	NETL[25,26]
Lifetime workovers_conventional&tight gas	times	0.0075	NETL[25,26]
Lifetime workovers_CBM&shale	times	0.125	NETL[25,26]
Other points sources emission rate_onshore	dimensionless	7.49E-05	NETL[42]
Other points sources emission rate_offshore	dimensionless	3.90E-05	NETL[42]
Leakage rate of valve	kg per hour	4.65E-03	NETL[43], EPA[44]
Leakage rate of connections	kg per hour	2.07E-04	NETL[45], EPA[44]
Leakage rate of OEL	kg per hour	2.07E-03	NETL[46], EPA[44]
Leakage rate of flange	kg per hour	4.03E-04	NETL[47], EPA[44]
Leakage rate of pneumatic device	standard cubic meter per hour	0.38	NETL[48], EPA[44]
Leakage rate of produced water tank	kg/m^3	8.08E-03	NETL[49]
Water produce per well	cubic meter per well	6.06E+04	NETL[49]
Other equipment leakage rate	%	0.024	NETL[50]
Inlet pressure at wellhead	Mpa	0.35	NETL[51]
Pressure required for gas gathering pipeline	Mpa	5.5	NETL[51]
Energy conversion factor of NG engine prime mover	dimensionless	3.11	OPGEE[28]
Energy conversion factor of NG turbine prime mover	dimensionless	3.62	OPGEE[28]
Energy conversion factor of electric prime mover	dimensionless	1.06	OPGEE[28]
Compressor efficiency	%	75	OPGEE[28]

Supplementary Table 5 Water content for major natural gas pipeline in China

Pipeline	Pipeline Water Content (mg/m3)
West-East Gas Pipeline	15
Shaan-Jing Gas Pipeline	53
Zhong-Wu Gas Pipeline	115
Gas pipeline of China National Offshore Oil Corporation	65
Sichuan-Shanghai Gas Pipeline	113

Supplementary Table 6 Key assumptions for calculating processing-associated emissions

Parameters	Unit	Value	Data sources
H_2S content in raw gas	vol%	Field-specific input	
CO_2 content in raw gas	vol%	Field-specific input	
With or without NGL separation	dimensionless	Field-specific input	
Amine reboiler heat duty	MJ/m^3 amine	279	NETL[52]
Amine reboiler heat efficiency	%	92	NETL[52]
CH4 absorbed rate of amine solution	kg CH_4/kg NG treated	9.72E-04	NETL[52]
Glycol (TEG) flow rate	m^3/tonne water	25	NETL[53]
TEG reboiler duty	MJ/m^3 TEG	313	NETL[53]
CH4 absorbed rate of glycol solution	kg CH_4/kg NG treated	3.4E-04	NETL[53]
Water content in raw gas	mg/m3	794	NETL[53]
Flaring rate	%	100	CPSC[54],NETL[25,26]
Flaring efficiency	%	98	IPCC[41]
Gas venting rate of other point sources	dimensionless	3.68E-04	NETL[55]
Leakage rate of pneumatic devices and vales	dimensionless	6.33E-06	NETL[56]
Leakage rate of other devices	dimensionless	8.25E-04	NETL[57]
Electricity input intensity of NGL separation	MWh/kg	1.38E-05	NETL[58]
Natural gas fuel input intensity of NGL separation	kg/kg	1.45E-06	NETL[58]

Supplementary Table 7 Key assumptions for calculating transmission-associated emissions

Parameters		Unit	Value	Data sources
Pipeline transmission distance		km	Field-specific input	
Share of different type of compressors used in the pipeline transmission in China	NG engine	%	1	Xu[59]
	NG turbine	%	83	
	Electric	%	16	
Energy intensity of pipeline transmission		MJ/tonne-km	1.19	GREET[60]
Pipeline leakage rate		kg/kg-km	3E-06	GREET[60], EPA[61], Brandt et al[62], NETL[25,26], Lelieveld et al[14], Alvarez et al[5], Dedikov et al[13]

Supplementary Table 8 Key parameters used to calculate GHG emissions of different liquefaction plants

Parameters	Unit	APLNG/ QCLNG	Gorgon LNG	PNG LNG	SPLNG	Qatargas LNG	Atlantic LNG	Nigeria LNG	Oman LNG	Snohvit LNG	Tangguh LNG
NG use intensity of refrigeration	MJ/kg NG	3.28	2.53	3.80	3.64	3.46	3.69	3.59	2.83	2.30	4.05
NG use intensity of generator turbine	MJ/kg NG	0.85	2.22	1.06	0.30	0.58	1.06	0.84	0.73	0.60	1.14
Diesel use intensity as backup power	MJ/kg NG	2.74E-04	0	0	0	0	0	0	0	0	1.06E-04
Equipment leakage rate	Dimensionless	8.63E-05	1.82E-03	0	3.33E-04	2.10E-04	2.10E-04	2.10E-04	7.44E-05	6.06E-05	4.18E-04
NG use intensity of heater/boiler	MJ/kg NG	0.06	0.01	0.48	0.00	0.03	0.07	0.03	0.06	0.05	0.11
Flaring NG	Dimensionless	4.04E-03	7.11E-04	0	6.62E-05	2.05E-03	4.51E-03	2.05E-03	3.48E-03	2.83E-03	2.91E-03
Data sources		ConocoPhillips[63], Chevron[64-66], Biswas et al[67], Houghton[68], Exxon Mobil[69], BP[70], Kewan Bond Pty Ltd[71], API[72], Nigeria LNG limited[73], Greencap[74], Barnett et al[75], SPL LLC[76], El-Houjeiri[77], Safaei[78], Schuller[79],Yost[80].									

Note: APLNG: Australia Pacific LNG, QCLNG: Queensland Curtis LNG, PNG LNG: Papua New Guinea LNG, SPLNG: Sabine Pass LNG. APLNG and QCLNG have long-term infrastructure sharing agreements, we use the same value for the two liquefaction plants[81].

Supplementary Table 9 Key assumptions for calculating emissions from LNG storage and shipping

Parameters	Unit	Value	Data sources
Ocean shipping distance (one way)	km	Field-specific input	
LNG boil-off rate	% per day	0.1	GREET[60]
Recovery rate of boil-off gas	%	80	GREET[60]
Days of storage at berth	days	5	GREET[60]
Days of storage at LNG receiving terminal	days	5	GREET[60]
Ocean tanker average speed	km/hour	32	GREET[60]
Energy conversion factor of ocean tanker	dimensionless	2.14	GREET[60]
Load factor from origin to destination	%	83	GREET[60]
Load factor back to origin	%	70	GREET[60]
Cargo payloads of LNG ocean tanker	tonne	59000	GREET[60]

Supplementary Table 10 Key assumptions for calculating emissions of LNG regasification

Parameters	Unit	Dapeng LNG	Dalian LNG-summer	Dalian LNG -winter	Dalian-average	China-average
Electricity use intensity	kWh/tonne LNG	23	26	14	20	21
NG use intensity	MJ/tonne LNG	0	0	489	245	122
Data sources	Chu et al[82], Li et al[83].					

Supplementary Note 1: Well-to-city-gate life-cycle assessment model of natural gas supply

Model structure and system boundary

For this study, we develop a Microsoft-Excel based model to estimate life cycle greenhouse gas (GHG) emissions of natural gas supply throughout well to city-gate. The life cycle assessment (LCA) of the study is in accordance with the International Organization for Standards (ISO) 14040 series standards.

Figure 6 in the main manuscript shows the system boundary and structure of the LCA model. The basic concept of the model is to use engineering-based models and field-specific parameters to estimate the energy and mass flow for gas supply from different fields, and the field-specific GHG emission intensity is calculated as the sum of emissions from all processes divided by the energy content of the final delivered gas. Noteworthy, we track the feedstock composition change from the gas flow out of the extraction well (field-specific input) to the end of supply chain. Feedstock loss and changes in compositions (thus the heating value) can be caused by multiple activities such as methane leakage, separation and venting of the CO_2 content from raw gas, and onsite gas combustion for heating and compression, etc. These activities and their impacts varied according to the operation of different gas fields and should be modeled in detail for analyzing the heterogeneity of GHG emissions intensity of different gas supplies.

Here, we will present the detailed methods and parameters used to calculate emissions from each process of natural gas supply.

Natural gas extraction

Well drilling

GHG emissions of well drilling arise from the emissions associated with diesel consumption of the drilling rig. The study uses the equation S1 to estimate the diesel consumptions of well drilling[40]:

$$FC = RC \times T = RC \times \frac{D}{PR} \quad [S1]$$

where, FC is the diesel consumption of drilling per well. RC is the drilling rig output power, which ranges from 1860 to 4920 kilowatt[40]. The drilling time T is calculated as the well measured depth D divided by the average penetration rate of the diesel drill PR, which varies from 14.6 to 16.8 meter per hour[40]. The measured depth D is the total length of the wellbore measured along the actual well path. For conventional vertical well, measured depth equals to the depth of the well (after subtracting water depth for offshore gas wells), while for the horizontal well of tight and shale gas extraction, measured depth D equals to well depth multiplied by the factor of 1.3 according to the practice experiences[40,84].

Diesel consumption of drilling one well is then divided by the estimated ultimate recovery (EUR) per well to calculate the diesel consumption rate of well-drilling. The onsite and upstream GHG emissions associated with well-drilling diesel consumption is calculated as the diesel consumption rate multiplied by the emission factor of diesel combustion onsite and offsite production and transportation.

Field-specific values of well depth and EUR are used in the calculation. The onsite and upstream emission factors associated with diesel are obtained from the GREET® 2018[60].

Well completion and well workover

Well completion is the preparation process for gas production after well drilling[25,26]. Well workovers are the occasional operations for well cleaning and maintenance that happen during the production life of well[25,26]. The study adopted the method from the National Energy Technology Laboratory's (NETL) natural gas LCA model to calculate the GHG emissions of well completion and well workover. For well completion of conventional and coal bed methane (CBM), the potential gas venting (before flaring) per well is estimated as 1.05 and 1.40 thousand cubic meter, respectively[25,26]. For tight and shale gas well, because higher potential emissions arise from the additional hydraulic fracking and higher flow-back period, the estimation of well completion emissions would have significant impacts on the final LCA results. Here, we considered the effects of individual heterogeneity and calculate the potential emissions of well completion of tight and shale gas as the field-specific initial production rate multiplied by the duration of flow-back period[25,26].

Not all wells require workover, while for some wells, workover can happen more than one time during the production life. The study adopts NETL's assumptions of average workover rate for different types of gas wells. The potential emissions of workover per episode for a conventional and CBM gas well are assumed as 0.069 and 1.40 thousand cubic meter, respectively[25,26]. The same estimation method is applied

to estimate the potential emissions for tight and shale gas as the product of the initial production rate and flow-back period.

The potential emissions of well completion and well workover are then divided by the field-specific estimated ultimate recovery (EUR) to calculate the potential emission rate of well completion and workover. The potential emissions will be further collected for flaring or just venting depending on the flaring and venting practice of the well, which will be discussed in the Section of Fugitive emission below.

Liquid uploading

Liquid uploading is the practice to remove water and other condensates from gas wells. Since there is no field-specific information about liquid uploading practices in China from both public and commercial database, we followed NETL's study of 2014 version to assume that one episode of liquid uploading released 102 cubic meter of natural gas, and a total of 930 unloadings happened over the well life[25,26]. The lack of field-specific data would increase the uncertainty of the final results considering the potential existing heterogeneity of liquid uploading among individual fields[85], which can be a research focus in the future study.

Similar as the emissions from well completion and workover, the potential emissions of liquid uploading can be collected for flaring, and the emission rate associated with liquid uploading is calculated as the episode emissions after flaring divided by field-specific EUR.

Fugitive emissions

Fugitive emissions include emissions from flaring, venting (purposeful release) and leakage (unpurposeful leaking)[41]. Episode emissions of well completion, well workover and liquid uploading, as well as routine emissions from point sources (e.g. heater) can be captured for flaring or just venting determined by the operation practice. Adapted from estimations by NETL[25,26] and IPCC[41], an average flaring rate of 51% for conventional gas and 15% for unconventional gas (tight, shale and CBM), and a flaring efficiency of 98% are used to calculate GHG emissions after flaring and venting for those point sources. Leakages at the extraction stage are unpurposeful leaking from well equipment including valve, connectors, flange, open-ended lines, pneumatic device, water tank, etc. Leakage associated with one type of device for a gas well production per day is calculated as the corresponding leakage rate of the device per hour multiple by the number of device connected and the total operational hours per day (24hr). Per day leakage of the device is then divided by the average production rate of the gas well to calculate the average leakage rate.

The average production rate of well is a field-specific parameter. The total leakage rate is the sum of leakage of all devices.

Gas gathering

At the wellhead, engine-driven compressors collect gas from multiple production wells and then transport gas to the processing facility. We follow NETL's assumption that the compressors' inlet and outlet pressures are 0.35 and 5.5 Mpa[25,26], respectively, and then use the engineering model from the Oil Production Greenhouse gas Emissions Estimator (OPGEE)[28] to estimate the energy consumption for gas gathering at the wellhead.

Key parameters used to calculate emissions associated with gas extraction stage are listed in Supplementary Table 4.

Natural gas processing

Acid gas removal

Acid gas removal (AGR) is a process to remove the H_2S and CO_2 content in the raw gas to meet the pipeline gas quality or liquefied natural gas (LNG) specification. The most common technique for acid gas removal is amine treating, which removes the H_2S and CO_2 by amine absorption and chemical reaction. After absorption, the amine solution would be sent to a reboiler to separate the acid gas and regenerate amine. The separated acid gas (with a portion of CH_4 absorbed from raw gas) is flared before venting to the atmosphere. GHG emissions of the acid gas removal process arise from the fuel consumption for reboiler and the residue gas after acid gas flaring. The fuel consumption of the amine reboiler is calculated by the parameter of heat duty, heat efficiency of the reboiler and the amount of amine consumed, which is determined by the acid gas content in the raw gas and the output gas quality requirement.

H_2S and CO_2 content of the raw gas are field-specific inputs for the calculation while the output gas quality requirement is determined by the industry standards. According to the Chinese standard[86], for pipeline gas, CO_2 content must be less than 3.0 vol% (equivalent to 0.47 wt%, which is the same as the U.S.' requirement[25,26]), and H_2S content must be less than 20 mg/m^3. LNG has a more strict specification of less than 0.01 vol% CO_2 and 0.0004% H_2S[87], for preventions of freezing and corrosion of the cryogenic liquefaction facilities.

We assume the reboiler use onsite-generated natural gas as fuel, and the GHG emission factor of natural gas combustion is then calculated based on its composition and carbon content, following the same calculation method in GREET®[60]. The consumption of onsite-generated natural gas also leads to feedstock loss which is tracked in the LCA model.

According to Chinese natural gas industry standards, the separated acid gas must be flared before venting to the atmosphere[54]. GHG emissions after flaring are calculated by the amount of CH_4 and CO_2 absorbed in the amine solution and the flaring efficiency.

Dehydration

Dehydration is the process to use glycol to absorb and remove the water content in the raw gas to meet the transportation and end-use requirement. Similar to the acid gas removal process, GHG emissions arise from the fuel consumption for the glycol-solution-reboiler and the residue gas after flaring.

The fuel consumption is a function of reboiler duty, glycol flow rate and the amount of water removed, which is determined by the mass balance of the raw gas water content and pipeline-required water content. We adopted NETL's assumption for the water content of raw gas as 794 mg/m^3. According to the Chinese natural gas industry standard, the pipeline-required water content is determined by the operational temperature and pressure of the connected pipeline of each gas field[86]. Supplementary Table 5 listed the pipeline water content requirement for major natural gas pipelines in China. The water content requirement for LNG is less than 0.1 ppm to avoid ice crystals from forming[87].

Natural gas liquid (NGL) separation

NGL separation is the process of removing the non-methane hydrocarbons of natural gas (also referred to as NGL). Not all natural gas processing plans have included the NGL separation and we include a parameter in the LCA model to determine whether the corresponding processing plant of a specific field has included NGL separation or not.

GHG emissions arise from the fuel (onsite-generated natural gas) and electricity input during the NGL separation. We adopted NETL's assumption on the electricity and fuel consumption rate for the NGL separation, and used emission factors from GREET® to convert energy consumptions to GHG emissions. Noteworthy, the life cycle GHG emissions factors of grid mix electricity for different gas production counties are different. Except for United States, China, Norway, Russia, Australia, of which the values are obtained from GREET® directly[60], GHG emission factors of electricity grid for other countries used

in the LCA model are the simulation results of applying each country's share of energy sources for power generation to the GREET® model[60]. Those countries' share of energy sources for power generation is obtained from International Energy Agency's World Energy Statistics (see Supplementary Data 4 for details)[88].

90% of C_2H_6 and 100% of C_3H_8 and C_4H_{10} are separated in the process[28,89], which are treated as bi-products for the LCA analysis. We allocate GHG emissions between well extraction and NGL separation to the main product (natural gas) and NGL according to their corresponding energy contents.

Fugitive emissions

Fugitive emissions of gas processing include emissions from flaring of the vented gas from AGR unit, dehydration unit, and other point sources, as well as leakage from gas processing equipment. The analysis of gas flaring of the AGR and dehydration units have been presented in sections above. Emissions from flaring of vented gas from other point sources (e.g., condensate tanks, blowdown, etc.) are estimated based on the factors of flaring rate and flaring efficiency, and the gas venting rate of these point sources, which is adopted from NETL's model (listed in Supplementary Table 6). We also adopted the leakage rates of gas processing devices from NETL's model to estimate the leakage emissions.

Gas compression

As mentioned above, we assume the pressure of gas gathering pipeline is 5.5 Mpa. Additional gas compression is required for gas fields connected with higher-pressure pipelines, such as the Chinese West-East gas pipeline, of which the operating pressure is 10 Mpa due to the extremely long-distance transmission. We used the calculation method from OPGEE[28] to estimate the energy consumption and GHG emissions associated with the additional gas compression.

Supplementary Table 6 listed the key parameters used to calculate emissions associated with gas processing.

Pipeline transmission

GHG emissions of pipeline transmissions arise from gas leakage and the energy consumption of compressors for the maintenance of gas pressure. We used the value of average energy intensity of pipeline transmission from GREET®[60] and field-specific pipeline transmission distance (see Supplementary Data 1) to calculate the energy consumption of pipeline transmission. GHG emissions associated with energy consumption is then calculated based on the share of different types compressor (i.e. natural gas (NG)

engine, NG turbine and electric) used for transmission and their corresponding emission factors. We assume NG engine and NG turbine compressors use the transported natural gas as fuel, therefore, the CO_2 emission factors of the compressors are calculated based on the carbon content of transported natural gas(estimated based on the tracked composition of feedstock flow) and the oxidation rate of the corresponding compressor fuel combustion, which is calculated using data from GREET®[60]. The life-cycle emission factor of electricity is obtained from GREET®[60] and varies according to the different electricity mix of the production countries, as mentioned above.

Leaking emissions are calculated based on the pipeline leakage rate and field-specific pipeline transmission distance. Pipeline leakage rate, despite its high uncertainty, is a key factor when calculating transmission-related emissions. We adopted the estimates for the United States and Russian pipeline systems available in previous studies given the lack of publicly available data for China's pipeline system[5,13,14,26,60-62]. The discussion about the impact of the uncertain pipeline leakage rate is presented in the section of Supplementary Discussion below.

Liquefaction

Natural gas liquefaction plant generally uses the feedstock natural gas as energy use in the facility, except for using diesel as back-up power in some cases. GHG emissions of liquefaction are derived from natural gas combustion for refrigeration compressor, generator turbines, heater and boilers, and fugitive emissions from flaring and equipment leakage[77]. Energy consumption rate varied for different liquefaction plants, and the ambient temperature is a key factor driving the differences. A low ambient temperature would facilitate the liquefaction process and improves energy efficiency. Supplementary Table 8 presented the energy consumption rate and fugitive emission rate for different LNG plants, which are estimated based on data obtained from a serial of publication including environmental assessment reports of LNG plants, industry reports and journal publications.

GHG emissions associated with the natural gas combustion are calculated based on the NG use intensity, the carbon content of the feedstock NG and the combustion oxidation rate of different type of compressors, following the similar calculation method in GREET®[60]. The amount of natural gas used for combustion is deducted from the feedstock before entering to the next processing unit.

Liquefied natural gas storage and shipping

GHG emissions associated with LNG storage and shipping derive from the LNG boil-off and fuel consumption of LNG shipping. The boil-off effect is the phenomenon when the ambient heat is input to the cryogenic fluid to create vapors. In the study, we followed the calculation method in GREET®[60] to calculate the fugitive emissions due to the LNG boil-off, which is the function of the factor of boil-off rate per day, recovery rate of boil-off gas, and the duration of storage and shipping. The duration of LNG ocean shipping is calculated basing on the shipping distance from each LNG exporting terminal to receiving ports in China.

We adopted the method in GREET®[60] to calculate the combustion emissions associated with fuel consumption of LNG ocean shipping. LNG ocean tanker uses the transported LNG as fuel for shipping from origin to destination, and use heavy fuel oil for shipping back to the origin. The shipping-associated emissions are calculated based on fuel consumption and the corresponding combustion emission factors of LNG and heavy fuel oil. Similar to the feedstock natural gas combustion scenarios mentioned above, the CO_2 emissions factor of the LNG fuel is calculated based on the carbon content of LNG. LNG combustion also leads to the feedstock loss, which is modeled specifically for each supply pathways of individual gas fields. The combustion and upstream emission factors of heavy fuel oil are obtained from GREET directly[60].

Key parameters used to calculate emissions associated with LNG storage and ocean shipping are presented in Supplementary Table 9.

Regasification

Limited studies have reported GHG emissions for LNG regasification process, and data from those studies are generally outdated and aggregated without complete details[90-92]. Here, we estimate the GHG emissions of regasification in China based on the energy consumption data obtained from case studies in China.

There are mainly two types of regasification technologies: the open rack vaporizers (ORVs) and the submerged combustion vaporizers (SCVs) employing in the LNG receiving terminals of China[93]. ORVs use seawater as the heating source to vaporize LNG, while SCVs use heats generated from natural gas combustion for the vaporization. The choices between the two technologies are mainly determined by the ambient temperature and the geographic location of the LNG terminal. According to the study by Agarwal

in 2017, 100% of the LNG terminals in China applied ORVs as the primary technique, while SCVs is the secondary technique when the ambient temperature is low[93]. For instance, Guangdong Dapeng LNG which locate in South China generally uses ORVs because the temperature of the ambient seawater is high enough for the entire year[82]. While Dalian LNG which locate in North China applies ORV for the summer mode and SCVs in winter when the ambient temperature is low[83]. We obtain the energy consumption data from two case studies of Guangdong LNG and Dalian LNG and use them as representatives for terminals located in the south and north China to calculate the average energy consumption rate for the LNG terminals in China. Supplementary Table 10 presents the estimated energy intensity value for LNG regasification. These values together with emission factors obtained from GREET[60] directly or calculated based on the LNG carbon contents are used to calculate the GHG emission of regasification.

Estimation of the missing value of fields-specific production profile

Field-specific production profile is important for the estimation of GHG emission intensity of different gas fields. In the study, EUR, average production rate and initial production rate (for tight and shale gas) are three field-specific production parameters input to the natural gas LCA model.

We gathered field-specific production data from industrial reports, journal publications, books, etc. Detailed data sources for each gas fields are presented in Supplementary Data 1. Although a wide range of publications has been searched, there are still multiple missing data in the production profiles. For the missing production data for gas fields outside of China, commercial data from Wood Mackenzie[94] are used to fill the gap, which is colored in orange in Supplementary Data 1. For Chinese domestic gas fields, due to the limited data in both public and commercial database, usually only initial production rate or the stable production rate at the early stage can be found, while the data for EUR and average production rate over the well life is incomplete. We use the following assumptions and methods to estimate the missing data of EUR and the average production rate for Chinese domestic natural gas fields:

Chinese conventional gas

According to the previous research[95] and management documentation released by China National Petroleum Corporation (CNPC)[96], the common production mode for a high permeability conventional gas well in China is to maintain a stable production rate for approximate 10 years (by controlling well pressures), and then the production rate declines till the end of well life. By assuming a 30-years of

production life, the EUR of a conventional natural gas well is thus estimated as the sum of cumulative gas production of the 10-years stable period and 20-years declining period. Arps's exponential production decline equations are used to estimate the cumulative production for the latter 20 years[97]:

$$q_t = q_i \exp(-Dt) \quad \text{[S2]}$$

$$Q = \frac{q_i}{D}[1 - \exp(-Dt)] \quad \text{[S3]}$$

where q_t is the production rate at the time of t for the decline period, which is the 10+t year from the beginning of well production. D is a constant parameter. q_i is the initial production rate. Q is the total cumulative production of the 20 years of the decline period. By assuming that the production rate at the end of well life is 0.01 of the initial production rate, EUR can be estimated as a function of the well's stable production rate at the early stage. And the average production rate per is calculated as the EUR divided by 30 years and 365 days.

Chinese unconventional gas

Wei et al. [98] studied the production characteristics for a typical shale gas well in Sichuan basin and a tight gas well in Ordos basin, which are the largest and most representative shale and tight gas production area in China (Sichuan and Ordos basin accounts for 99.8% and 83.2% of the total shale gas and tight gas production in China in 2016, respectively). According to the analysis, a typical shale gas well in Sichuan basin has a production life of ~30 years, and a EUR of ~ 80 million cubic meters. A typical tight gas well in Ordos basin has a production life of ~20 years, and a EUR of ~ 65 million cubic meter[98]. Adopting the method by Energy Research Institute of National Development and Reform Commission (ERINDRC)[99] and Qin[100], we assumed that the production curve of other shale gas wells in China have a similar shape as that for the typical shale gas well in Sichuan basin, and the missing EUR for other shale gas wells can be estimated by scaling the EUR of the typical shale gas well:

$$\frac{EUR_{other\ shale\ gas\ well}}{EUR_{Sichuan\ typical}} = \frac{Initial\ production_{\ other\ shale\ gas\ well}}{Initial\ production_{Sichuan\ typical}} \quad \text{[S4]}$$

$$EUR_{other\ shale\ gas\ well} = EUR_{Sichuan\ typical} \times \frac{Initial\ production_{\ other\ shale\ gas\ well}}{Initial\ production_{Sichuan\ typical}} \quad \text{[S5]}$$

Therefore, the missing EUR value of other shale gas wells can be calculated with the field-specific initial production rate. And the missing average production rate is calculated as the EUR divided by the total production days of the well life. A similar method is used to estimate the missing EUR and average production rate of tight gas wells in China by scaling the production data of the typical tight gas well in Ordos basin.

The estimated value of EUR and the average production rate for Chinese domestic gas wells are colored with green in Supplementary Data 1.

Supplementary Discussion 1: Sensitivity analysis

Due to the uncertainty of parameters input to the well-to-city-gate natural gas LCA model, we conduct a sensitivity analysis to exam how the uncertain input would affect the robustness of results. The well-to-city-gate natural gas LCA model contain 88 uncertain input parameters, including factors with heterogeneity of individual gas fields (e.g. raw gas composition, EUR, pipeline transmission distance, etc), as well as those are not varied with gas fields (e.g. compressor prime mover fuel use rate, boiler heat efficiency, LNG boil-off rate, etc). We vary one parameter each time while maintaining other parameters constant to evaluate the sensitivity of each parameter on the estimation of GHG emission intensity for different gas fields. For each simulation, we increase the value of a parameter by 20% unless there is a fixed upper limit for the parameter and then decrease 20% instead (e.g., 100% is the upper limit for flaring rate). To facilitate the comparison and identifications of the sensitive parameters, we calculate the elasticity of each parameter *i* to the estimation of the GHG emission intensity of gas filed *j*:

$$E_{i,j} = \frac{\Delta y_j}{\Delta x_i} \cdot \frac{x_i^0}{y_j^0} = \frac{\widehat{y_j} - y_j^0}{\widehat{x_i} - x_i^0} \cdot \frac{x_i^0}{y_j^0} = \frac{\%\ change\ in\ y_j}{\%\ change\ in\ x_i} \quad [S6]$$

where, y_j is the GHG emission intensity of gas field j, x_i is the value of parameter i. y_j^0 and $\widehat{y_j}$ represent the estimation of GHG emission intensity for gas field *j* before and after the variation of parameter x_i. x_i^0 and $\widehat{x_i}$ represent the value input of parameter x_i before and after variation.

In general, parameters' sensitivity varies for different gas fields while gas fields of the same category show a certain similarity. Technical parameters of compressors (e.g. compressor efficiency, prime mover fuel use rate of compressor engine) are sensitive parameters for gas fields of all categories because of the

extensively used and energy-intensive of compressors throughout the natural gas supply chain. Parameters associated with pipeline transmissions (i.e. pipeline transport distance, energy intensity rate of pipeline transmission, pipeline leakage rate) are sensitive for carbon intensity estimation of pipeline sources gas fields, especially for international gas fields and domestic gas fields with extremely long transmission distance (e.g. Dina, Kela, Yakela, Yingmaili, Tazhong gas fields). The parameter of pipeline transport distance has the highest elasticity (~1.1) on the estimation of emission intensity for pipeline gas supply from Turkmenistan, Uzbekistan and Russia. The parameter of CO_2 composition in raw gas is among the highest sensitive parameters for gas fields with high CO_2 content (e.g. Dongfang, Ya, Wenchang, Liuhua, Indonesia Tangguh gas fields). CO_2 composition in raw gas has the highest elasticity (0.87) to the estimate of Dongfang gas field. For domestic unconventional tight and shale gas, because fugitive emissions from well completion accounts for a significant portion in the overall emissions, parameters used to calculate the emission rate of well completion (such as initial production rate, flow-back period, EUR, flaring rate and flaring efficiency) are all highly sensitive to the final results. For international LNG, parameters associated with the liquefaction process (i.e. fuel use of refrigeration compressor, fuel use of generator turbines at LNG plants) and LNG storage and shipping (i.e. LNG boil-off rate, recovery rate of boil-off gas, LNG ocean shipping distance, ocean tanker average speed) are sensitive to the estimation of GHG emission intensity.

For most of the sensitive parameters mentioned above, we have conducted detailed analysis and identified the heterogeneity of parameters among individual gas fields, which would significantly reduce the uncertainty of the LCA results. For instance, raw gas composition, initial production rate, EUR, pipeline transport distance are field-specific input to the LCA model. These highly-sensitive and individual-specific parameters contribute most to the variability of emission intensity among different gas fields. Other parameters, for instance, compressor efficiency, energy intensity rate of pipeline transmission, pipeline leakage rate, LNG boil-off rate and recovery rate of boil-off gas, are set as constant for estimations across gas fields because no sufficient data suggests their variation among individual gas fields. Although we have referred to established model (e.g. GREET model, NETL's natural gas LCA model) and reliable data sources (e.g. EPA GHG inventory) to estimate the value of these parameters, the uncertainty inevitably exists in these parameters and the final LCA results. In the next section, we will further explore the uncertainty ranges of these parameters and thus estimate the overall uncertainty in the final LCA

results. Tornado diagrams in Supplementary Fig. 4-7 show the variation of carbon intensities derived from uncertain inputs of top 5 parameters for four typical gas fields of the four gas categories

Supplementary Discussion 2: Uncertainty analysis

The above sensitivity analysis has identified parameters that have significant effects on the final results. In this section, we will estimate the probability distributions for those sensitive parameters and then conduct Monte Carlo simulations to calculate the uncertainties of final results.

Uncertainty of pipeline leakage rate

Leakage rate of pipeline transmission is a key factor to determine the transmission-associated and the overall emissions, especially for gas sourced from long-distance international pipelines. Enormous research efforts have been done on the measurements of pipeline system leakages since the 1990s[2-5,13-15,30,39,101-110]. Yet there is still high uncertainty and debate on this issue[39,62,105,106].

To have a clear understanding of the uncertainty of pipeline leakage rate and its impacts on our final results, here, we conduct a literature review on the pipeline leakage studies and incorporate the uncertainty of the pipeline leakage rate into the Monte Carlo simulation to generate robust results.

The majority of studies on pipeline transmission leakages were centered on the U.S. and Russia, which are the two largest natural gas producers that account for ~40% of gas production worldwide[39]. Pipeline leakage rates are also measured and reported in some European countries, such as United Kingdom, Germany and the Netherlands[30,104], but since China does not import natural gas from these European countries, studies in those countries will not be discussed here. There is no publicly available report on the leakage rate of China's pipeline system.

Supplementary Fig. 3 summarizes literature estimates of the pipeline leakage rate for the U.S. and Russia. Noteworthy, we have converted the percentage leakage rate of throughput to the leakage rate on per kilometer (km) of transport distance basis. The lengths of the U.S and Russian pipeline system that used for conversions are obtained from the literature. Studies of Russia's pipeline system leaks are focused on Russia's export corridors to Western European. The length of the export corridors is ~3000km within the Russian border (the distance of Northern Corridor is 3075km while the Central Corridor is 3376km)[13,15]. The average transmission distance of the U.S natural gas system was estimated at ~1000km[25,26,60,111]. The much longer transmission distance of Russia's natural gas system leads to its higher leakage rate in terms of percentage throughput than that of the U.S. system in literature[14,15,39]. Yet on the per km basis, pipeline

leakage rates of the two systems are comparable and their uncertainty ranges overlap mostly, as shown in Supplementary Fig. 3.

The difference in the estimations of pipeline leakage rate is caused by many reasons. Firstly, varied measurement approaches would result in different estimates[62]. There are generally two types of measurement approaches: the top-down and the bottom-up approaches. Top-down studies use aircraft, tower, remote sensing, etc. to measure the concentrations of CH_4 or other volatile organic compounds (VOCs) in the atmosphere[5,39,62]. The atmospheric observations are then attributed to different sources, including multiple anthropogenic sources (such as waste landfill, production of ruminant animals, oil, coal and natural gas production, etc) and natural sources (such as natural wetland, natural geologic seeps, etc.)[12,62]. The sources attribution is the key challenge for the top-down study and it will introduce significant uncertainties since assumptions about the activity levels of different sources are highly uncertain[62].Bottom-up studies measure emissions of sampling devices or facilities, and then multiple the emission factors by device counts or activity factors to make emissions inventories[5-12,62]. According to the literature review by Brandt et al, inventories generated from bottom-up studies, such as the U.S. Environmental Protection Agency (EPA) GHG inventory (GHGI), systematically reported methane emissions lower than top-down measurements[62]. Limited sample sizes and representatives, uncaptured "superemitters", and incomplete statistics of activities and devices are responsible for the potential underestimates of bottom-up studies[39,62]. Supplementary Fig. 3 includes estimates from both top-down and bottom-up studies, which should have captured the uncertainties from different measurement approaches.

Secondly, characteristics of the pipeline systems, such as pipeline age, operating pressure, level of maintenances and mitigation practices, would affect the pipeline leakage rates[14,38,39,109,110]. Previous researches revealed that old compressor stations might lead to higher leaks, and pipeline system with higher operating pressures has the tendency to increase leaks, while well maintenance and mitigation measures can significantly reduce transmission leakages[14,38,39,109,110].

Supplementary Table 1 compares the characteristics of China's pipeline system with those of the U.S. and Russia to look for clues about the level of China's transmission leakage rate. As shown in Supplementary Fig. 3, compared to U.S and Russia, China has a much younger pipeline system which might help reduce leaks while a relatively higher operating pressure (10 MPa for the major gas pipelines such as West-East gas pipeline and Central-Asia gas pipeline), which, on the other hand, would potentially increase leaks.

Methane emissions are not regulated and reported in China and the country does not actively promote emissions mitigation practices in its pipeline system operations. In contrast, U.S. and Russia have promoted and deployed multiple methane emission mitigation measurements, such as forward line pumping, corrosion repair, replacement of high-bleed pneumatic devices, targeted inspection, etc, which are considered to help significantly reduce transmission leaks[39,107-109]. Kiefner and Rosenfeld studied pipeline incidents (including leaking seals and corrosions that are related to gas leaks) reported to U.S. Pipeline and Hazardous Material Safety Administration (PHMSA) during 2002~2009 and found that 85% of the pipeline incidents are in irrespective of the pipeline age, with just 15% of the incidents related in some way to the pipeline age, and they concluded that periodically assessment, timely repairs with mitigation efforts can ensure aged pipeline's continued fitness for service[38].

After carefully comparing the characteristics of the Chinese pipeline system to those of the U.S. and Russia, we are not expecting the Chinese pipeline leakage rate to be significantly higher or lower than that of the U.S or Russia. Actually, literature estimates of pipeline leakage rates for the U.S. and Russia have wide uncertainty ranges, which should be large enough to capture the possible value for the Chinese pipeline leakage rate. According to Lelieveld et al, the true value of leakage rate for Russia's gas export transmission system must be lower than their upper estimates of 1.6% (~7.1E-06 kg/kg-km, the highest estimate for Russia in Supplementary Fig. 3), because the upper estimation is calculated based on "worst-case assumptions" in numerous areas[14]. Balcombe et al believed that estimates of the leakage rate of pipeline transmission (exclude leakages from production and processing) above 1.6% are results of outdated data or flawed estimation methods[39].

In the study, we apply the uncertainty estimates of Russia's pipeline leakage rate (which has a larger uncertainty range than that of U.S, as shown in Supplementary Fig. 3) to that of the Chinese pipeline system and the connected international pipeline system, such as the Central Asia-China pipeline, and conduct Monte Carlo simulation for uncertainty analysis.

Uncertainty of other sensitive parameters

Besides the transmission leakage rate, variations of some other parameters also have significant impacts on our LCA results. According to previous sensitivity analysis, parameters with elasticity greater than 0.1 (which means the final results would change more than 1% with 10% change in the parameter) include: CO_2 content in raw gas, average production rate, initial production rate, EUR, pipeline transport distance, flaring rate at extraction stage, well completion flowback period, compressor efficiency, energy

conversion factor of NG engine prime mover, energy conversion factor of NG turbine prime mover, flaring efficiency, energy intensity rate of pipeline transmission, LNG boil-off rate, recovery rate of boil-off gas, ocean tanker average speed. Among these parameters, CO_2 content in raw gas, average production rate, initial production rate, EUR, pipeline transport distance are field-specific inputs, of which the individual variations have been analyzed thoroughly. Here we assumed ±10% variation for these field-specific inputs, except for the EUR, of which ±50% of variations have been assumed because of the relatively high uncertain feature of the parameter. For other sensitive parameters, we estimate their uncertain range through literature reviews, as shown in Supplementary Table 2.

Monte Carlo simulation

With the probability distributions of the sensitive parameters, we conducted 5000 times Monte Carlo simulations to calculate the uncertainty ranges of the final results. Well-to-city-gate GHG intensities with 90% confidence interval (CI) for each gas fields are presented as error bars in Fig.1, Fig.2, Fig. 4 in the main manuscript and in Supplementary Fig. 1, 10 and 11. Supplementary Fig. 8 and 9 show the probability density function (PDF) of the well-to-city-gate GHG intensity of gas supply from Galkynysh field.

Supplementary Discussion 3: Economic analysis

The present study focuses on providing climate-wise choices for China to minimize GHG emissions for its growing natural gas supply. One important question remains regarding the economic cost and its effects on China's choice of global natural gas consumption. In the section, we compare the cost of supply for different gas sources and thus provide further insight into the economic implications of China's natural gas supply policy.

Overseas liquefied natural gas

The cost of overseas LNG supply includes the import price and other additional costs, such as the cost of regasification[23,112]. The LNG import price accounts for more than 95% of the total supply cost of overseas LNG[23,112]. The LNG import price is positively related to the global oil price and also affected by the market supply and demand conditions[113]. The growth of Spot LNG price can break through the trend of global oil price when there are strong demand and tight supply in the LNG market. Impacted by the fluctuating oil price and uncertain market conditions, LNG import price varies in a wide range. As shown

in Supplementary Fig. 12, from January to October 2019, China's LNG import price varied between 280~420 us dollar (USD) per thousand cubic meters (kcm, on the basis of gas volume after regasification)[22].

International pipeline gas

The import price of international pipeline gas is determined by the pricing formula in the long-term contract, which has never been disclosed by China National Petroleum Corporation. According to the published statistics of the import prices of international pipeline gas in recent years[114,115], the price of international pipeline gas has positive relationship with the global oil price and an extra price increase poses when there are tight supply and strong demand. Generally speaking, the average import price of pipeline gas is 20%~30% lower than that of overseas LNG during the same period[113-115]. However, since the international pipeline gas is mainly imported from the western border that is distant from the eastern coastal metropolitan areas, the extra cost of long-distance pipeline transmission significantly increases the total cost of supply. According to the study by Rioux et al[112], after including the cost of delivering gas across ~4000 km, the total cost of pipeline gas imports from Central Asia to Shanghai is similar to the coastal LNG import price. Supplementary Table 3 shows the import price of international pipeline gas in 2018 and the estimations of gas delivering cost within China for different import sources.

Domestic gas

Supply cost for domestic gas is relatively low but its production capacity is limited and far below China's prospective gas demand in the future[23,116,117]. The average production cost of domestic conventional gas varies between 20~110 USD/kcm in different provinces[23,112], as shown in Supplementary Fig. 13. Even with the cost of transmission, the total cost of domestic conventional gas supply is still much lower than the average import price of LNG in recent years[114,115]. China is now actively promoting the exploration of unconventional gas, such as shale gas in Sichuan Basin[100,118-120]. The production cost of shale gas in literature is estimated to be 130~400 USD/kcm[23,24,112]. With the cost of transmission, the total cost of domestic shale gas can be close to the gas import price.

Supplementary Fig. 14 compares the supply costs of different sources of gas supply to Shanghai. Due to the lack of public data from natural gas production sector and the involving international gas market, high uncertainty exists in the estimations of natural gas supply costs.

The comparison of supply costs for different categories of gas sources varies for different regions of China owing to the different production cost of domestic gas and relatively transport distance of gas imports. More in-depth economic analysis is required regarding the heterogeneity between different gas sources and different regions of gas consumption, which can be the focus for our future studies.

Supplementary Reference

1 National Bureau of Statistics of China & National Energy Administration of China. *China Energy Statistical Yearbook 1991-2017*. (China Statistics Press, 1991-2017).

2 Zimmerle, D. J. *et al.* Methane emissions from the natural gas transmission and storage system in the United States. *Environmental science & technology* **49**, 9374-9383 (2015).

3 Logan, J. *et al.* Natural gas and the transformation of the US energy sector: electricity. (National Renewable Energy Lab.(NREL), Golden, CO (United States), 2012)*;*

4 Laurenzi, I. J. & Jersey, G. R. Life cycle greenhouse gas emissions and freshwater consumption of Marcellus shale gas. *Environmental science & technology* **47**, 4896-4903 (2013).

5 Alvarez, R. A. *et al.* Assessment of methane emissions from the US oil and gas supply chain. *Science* **361**, 186-188 (2018).

6 Inventory of US greenhouse gas emissions and sinks: 1990–2011. (United States Environmental Protection Agency (USEPA), Washington DC, 2013)*;*

7 Inventory of US greenhouse gas emissions and sinks: 1990–2012. (USEPA, Washington DC, U.S., 2014)*;*

8 Inventory of us greenhouse gas emissions and sinks: 1990-2013. (USEPA, Washington, DC, 2015)*;*

9 Inventory of US greenhouse gas emissions and sinks: 1990-2014. (USEPA, Washington, DC, 2016)*;*

10 Inventory of US greenhouse gas emissions and sinks: 1990–2015. (USEPA, Washington, DC, 2017)*;*

11 Inventory of US Greenhouse Gas Emissions and Sinks: 1990–2016. (USEPA, Washington, DC, 2018)*;*

12 Inventory of US greenhouse gas emissions and sinks: 1990–2017. (USEPA, Washington, DC, 2019)*;*

13 Dedikov, J. *et al.* Estimating methane releases from natural gas production and transmission in Russia. *Atmospheric Environment* **33**, 3291-3299 (1999).

14 Lelieveld, J. *et al.* Greenhouse gases: Low methane leakage from gas pipelines. *Nature* **434**, 841 (2005).

15 Lechtenböhmer, S. *et al.* Greenhouse gas emissions from the Russian natural gas export pipeline system. *Wuppertal/Mainz: Wuppertal Institute and Max Planck Institute* (2005).

16 National inventory report: anthropogenic emissions by sources and removals by sinks 1990-2012. (Russian Federation Moscow, 2014)*;*

17 National inventory report: anthropogenic emissions by sources and removals by sinks 1990-2012. (Russian Federation, Moscow, 2015)*;*

18 National inventory report: anthropogenic emissions by sources and removals by sinks 1990-2014. (Russian Federation, Moscow, 2016)*;*

19 National inventory report: anthropogenic emissions by sources and removals by sinks 1990-2015. (Russian Federation Moscow, 2017)*;*

20 National inventory report: anthropogenic emissions by sources and removals by sinks 1990-2016. (Russian Federation, Moscow, 2018)*;*

21 Bulletins Environmental Protection: Information on air quality in 2015. (Russian Federation Federal State Statistics Service (Rosstat), 2015)*;*

http://www.gks.ru/wps/wcm/connect/rosstat_main/rosstat/ru/statistics/publications/catalog/5e901c0042cb5cc99b49bf307f2fa3f8
22 *Shanghai Petroluem and Gas Exchange* (2019); https://www.shpgx.com/html/index.html
23 Zhang, Q., Li, Z., Wang, G. & Li, H. Study on the impacts of natural gas supply cost on gas flow and infrastructure deployment in China. *Applied energy* **162**, 1385-1398 (2016).
24 Michael F. Farina, A. W. China's age of gas: Innovation and change for energy development. (General Electric Company, 2013);
25 Littlefield, J. *et al.* Life cycle analysis of natural gas extraction and power generation. (National Energy Technology Laboratory, 2014); https://www.netl.doe.gov/projects/files/NaturalGasandPowerLCAModelDocumentationNG%20Report_052914.pdf
26 Skone, T. J. *et al.* Life cycle analysis of natural gas extraction and power generation. (National Energy Technology Laboratory 2016); https://www.netl.doe.gov/projects/files/LifeCycleAnalysisofNaturalGasExtractionandPowerGeneration_083016.pdf
27 Simpson, D. A. in *Practical Onshore Gas Field Engineering* 513-571 (Gulf Professional Publishing, 2017).
28 El-Houjeiri, H., Vafi, K., Duffy, J., McNally, S., & Brandt, A. R. . *Oil Production Greenhouse Gas Emissions Estimator. OPGEE version 2.0: Computer program.* (2017); https://eao.stanford.edu/research-areas/opgee
29 Willis, J. *et al. Flare efficiency estimator and case studies*. (Iwa Publishing, 2014).
30 Müller-Syring, G., Große, C. & Glandien, J. Critical evaluation of default values for the GHG emissions of the natural gas supply chain. *Leipzig, Germany: DBI Gas-und Umwelttechnik GmbH* (2016).
31 Dobrota, Đ., Lalić, B. & Komar, I. Problem of boil-off in LNG supply chain. *Transactions on maritime science* **2**, 91-100 (2013).
32 Głomski, P. & Michalski, R. Problems with determination of evaporation rate and properties of boil-off gas on board LNG carriers. *Journal of Polish CIMAC* **6**, 133-140 (2011).
33 Sedlaczek, R. Boil-Off in Large and Small Scale LNG Chains. *MS Pet Eng Dep Pet Eng Appl Geophys Nor Univ Sci Technol Trondheim* (2008).
34 Kwak, D.-H. *et al.* Energy-efficient design and optimization of boil-off gas (BOG) re-liquefaction process for liquefied natural gas (LNG)-fuelled ship. *Energy* **148**, 915-929 (2018).
35 Smith, T. *et al.* Third IMO GHG Study. (International Maritime Organization (IMO), London, United Kingdom, 2015);
36 Buhaug, Ø. *et al.* Second IMO GHG Study 2009. (International Maritime Organization (IMO), London, United Kingdom, 2009);
37 Folga, S. Natural gas pipeline technology overview. (Argonne National Lab.(ANL), Argonne, IL (United States), 2007);
38 Kiefner, J. & Rosenfeld, M. The role of pipeline age in pipeline safety. *Interstate Natural Gas Association of America (INGAA)* (2012).
39 Balcombe, P., Anderson, K., Speirs, J., Brandon, N., and Hawkes A. Methane & CO2 emissions from the natural gas supply chain report. (Sustainable Gas Institute,Imperial College London, 2015);
40 Jiang, M. *et al.* Life cycle greenhouse gas emissions of Marcellus shale gas. *Environmental Research Letters* **6**, 034014 (2011).
41 2006 IPCC Guidelines for National Greenhouse Gas Inventories, Volume 2 Chapter 4 Fugitive Emissions. (Intergovernment Panel on Climate Change(IPCC), 2006); https://www.ipcc-nggip.iges.or.jp/public/2006gl/pdf/2_Volume2/V2_4_Ch4_Fugitive_Emissions.pdf

42 NETL Life Cycle Inventory Data – Natural Gas Extraction, Other Venting Point Sources. . (National Energy Technology Laboratory); www.netl.doe.gov/energyanalyses (http://www.netl.doe.gov/energy-analyses)
43 NETL Life Cycle Inventory Data – Unit Process: Natural Gas Extraction Fugitive Emissions - Valves., (National Energy Technology Laboratory, 2015); www.netl.doe.gov/LCA
44 Oil and Gas Emission Estimation Tool. (United States Environmental Protection Agency, 2013); www.epa.gov/ttn/chief/net/2011inventory.html
45 NETL Life Cycle Inventory Data – Unit Process: Natural Gas Extraction Fugitive Emissions - Connections. . (National Energy Technology Laboratory, 2015); www.netl.doe.gov/LCA
46 NETL Life Cycle Inventory Data – Unit Process: Natural Gas Extraction Fugitive Emissions - OEL., (National Energy Technology Laboratory, 2015); www.netl.doe.gov/LCA
47 NETL Life Cycle Inventory Data – Unit Process: Natural Gas Extraction Fugitive Emissions - Flanges. . (National Energy Technology Laboratory, 2015); https://www.netl.doe.gov/energy-analyses/temp/DF_Stage1_O_NG_Extraction_Fugitive_Flange_2015.01.pdf
48 NETL Life Cycle Inventory Data – Unit Process: Natural Gas Production Pneumatic Device Venting. (National Energy Technology Laboratory, 2015); www.netl.doe.gov/LCA
49 NETL Life Cycle Inventory Data – Unit Process: Natural Gas Extraction Produced Water Tank Venting. . (National Energy Technology Laboratory, 2014); https://www.netl.doe.gov/energy-analyses/temp/DF_Stage1_O_NG_Extraction_ProducedWaterTank_Venting_2014.01.pdf
50 NETL Life Cycle Inventory Data – Natural Gas Extraction, Other Venting Fugitives., (National Energy Technology Laboratory, 2010); http://www.netl.doe.gov/energy-analyses
51 NETL Life Cy cle Inventory Data – Unit Process: Wellhead Compressor, Gas - Powered Centrifugal, 200 HP. (National Energy Technology Laboratory, 2011); www.netl.doe.gov/energy-analyses
52 NETL Life Cycle Inventory Data – Unit Process: Natural gas sweetening. (National Energy Technology Laboratory, 2010); www.netl.doe.gov/energy-analyses
53 NETL Life Cycle Inventory Data – Unit Process: Natural Gas Dehydration. (National Energy Technology Laboratory, 2011); https://www.netl.doe.gov/energy-analyses/temp/DF_Stage1_O_NG_Dehydration_2011-01.pdf
54 Code for design of natural gas conditioning plant: SY/T 0011-2007 (China Petroleum Standardization Committee, 2008);
55 NETL Life Cycle Inventory Data – Natural Gas Processing, Other Venting Point Sources. (National Energy Technology Laboratory, 2011); https://www.netl.doe.gov/energy-analyses/temp/DF_Stage1_O_NG_Processing_OtherVenting_PointSource_2011-01.pdf
56 NETL Life Cycle Inventory Data – Natural Gas Processing, Pneumatic Venting. (National Energy Technology Laboratory, 2011); https://www.netl.doe.gov/energy-analyses/temp/DF_Stage1_O_NG_Processing_PneumaticVenting_2011-01.pdf
57 NETL Life Cycle Inventory Data – Natural Gas Processing, Other Venting Fugitives. (National Energy Technology Laboratory, 2011); https://www.netl.doe.gov/energy-analyses/temp/DF_Stage1_O_NG_Processing_OtherVenting_Fugitives_2011-01.pdf
58 NETL Life Cycle Inventory Data – Unit Process: Natural Gas Liquid Separation. (National Energy Technology Laboratory, 2015); www.netl.doe.gov/LCA
59 Xu, T. Application and Development of Natural Gas Pipeline Compressors in China (In Chinese). *Oil & Gas Storage and Transporatation* **5**, 321-326 (2011).
60 Wang, M. *The Greenhouse Gases, Regulated Emissions, and Energy Use in Transportation (GREET) Model* (2018); https://greet.es.anl.gov/

61 Inventory of US greenhouse gas emissions and sinks 1990-2017 (Office of Policy US Environmental Protection Agency, Planning, and Evaluation, 2018); https://www.epa.gov/sites/production/files/2019-04/documents/us-ghg-inventory-2019-main-text.pdf
62 Brandt, A. R. *et al.* Methane leaks from North American natural gas systems. *Science* **343**, 733-735 (2014).
63 Australia Pacific LNG Project Environmental Impact Statement (ConocoPhillips, 2010); https://www.aplng.com.au/about-us/compliance/eis.html
64 Final Environmental Impact Statement/Response to Submissions on the Environmental Review and Management Programme for the Proposed Gorgon Development (Chevron, 2006); https://australia.chevron.com/-/media/australia/our-businesses/documents/entire_document.pdf
65 Gorgon Gas Development and Jansz Feed Gas Pipeline (Chevron, 2014); https://ace.dmp.wa.gov.au/ACE/Public/PetroleumProposals/ViewPlanSummary?registrationId=45198
66 Shannon, K. Gorgon Upstream Overview. (Chevron, 2012); http://phx.corporate-ir.net/External.File?item=UGFyZW50SUQ9MTU0NDk5fENoaWxkSUQ9LTF8VHlwZT0z&t=1
67 Biswas, W., Engelbrecht, D. & Rosano, M. A life cycle assessment of Western Australian LNG production and export to the Chinese market. (Sustainable Engineering Group, Curtin University Perth, Australia, 2011); https://pdfs.semanticscholar.org/b177/683782e5ae60ba80661b837930623d3c37f4.pdf
68 Daryl J Houghton, L. S. R. The new age of LNG projects-Gorgon LNG. (1998); http://www.ivt.ntnu.no/ept/fag/tep4215/innhold/LNG%20Conferences/1998/Papers/4-6-Houghton.PDF
69 PNG LNG Project Environment Impact Statement. (Exxon Mobil Corporation, 2008); https://pnglng.com/media/PNG-LNG-Media/Files/Environment/EIS/eis_chapter01.pdf
70 Watkins, P. The Papua New Guinea LNG project. (BP Exploration, 1998);
71 Papua New Guinea Liquefied Natural Gas Project: Greenhouse Gas Assessment (Kewan Bond Pty Ltd, 2008); https://pnglng.com/media/PNG-LNG-Media/Files/Environment/EIS/eis_appendix25.pdf
72 Liquefied natual gas (LNG) operations: Consistent methodology for estimating greenhouse gas emissions. (American Petroleum Institute, 2015); https://www.api.org/~/media/Files/EHS/climate-change/api-lng-ghg-emissions-guidelines-05-2015.pdf
73 Facts and figures on NLNG 2014. (Nigeria LNG Limited, 2014); http://www.nlng.com/Media-Center/Publications/2014%20NLNG%20FACTS%20AND%20FIGURES.pdf
74 Tangguh Expansion Project Environmental and Social Impact Assessment Report. (Greencap, 2016); https://www.adb.org/sites/default/files/project-document/212941/49222-001-esia-03.pdf
75 Barnett, P. J. *Life Cycle Assessment (LCA) of Liquefied Natural Gas (LNG) and its environmental impact as a low carbon energy source*, University of Southern Queensland, (2010).
76 Environmental Assessment for the Sabine Pass Liquefaction Project (Federal Energy Regulatory Commission, 2011); https://www.energy.gov/sites/prod/files/EA-1845-FEA-2011.pdf
77 El‐Houjeiri, H., Monfort, J. C., Bouchard, J. & Przesmitzki, S. Life Cycle Assessment of Greenhouse Gas Emissions from Marine Fuels: A Case Study of Saudi Crude Oil versus Natural Gas in Different Global Regions. *Journal of Industrial Ecology* **23**, 374-388 (2019).
78 Safaei, A., Freire, F. & Henggeler Antunes, C. Life-cycle greenhouse gas assessment of Nigerian liquefied natural gas addressing uncertainty. *Environmental science & technology* **49**, 3949-3957 (2015).
79 Schuller, O. *et al.* Greenhouse gas intensity of natural gas. *Thinkstep AG, Natural & Bio Gas Vehicle Association (NGVA) Europe*, 180 (2017).
80 Yost, C. & DiNapoli, R. Benchmarking study compares LNG plants costs. *Oil & gas journal* **101**, 56-56 (2003).

81 Australia Pacific LNG to share infrastructure and secure additional gas supply to diversify portfolio (Australia Pacific LNG, 2018); https://www.aplng.com.au/content/dam/aplng/media-release/2018/APLNG%20Media%20Release%20-%20QCLNG%20agreement%20-%2020181105%20-%20FINAL.pdf
82 Yanqun Chu, W. C., Junfeng Niu, Xinling, Liu. LNG receiving terminal application technology (I) (In Chinese). *Industry of Natural Gas* (2006).
83 Xin Li, S. C. Calulating Energy Consumption of Vaporization Unit in LNG Receiving Terminal (In Chinese). *Chemical Engineering of Oil & Gas* **45**, 109-116 (2016).
84 Development example of Weiyuan shale gas, in US-China shale gas workshops 2017. (Research Institute of Petroleum Exploration and Development of PetroChina, 2017); https://www.gti.energy/training-events/events-overview/
85 Zaimes, G. G. *et al.* Characterizing Regional Methane Emissions from Natural Gas Liquid Unloading. *Environmental Science & Technology* **53**, 4619-4629 (2019).
86 Natural gas: GB/T 17820-2012. (General Administration of Quality Supervision, Inspection and Quarantine of the People's Republic of China & Standardization Administration of the People's Republic of China Beijing, China, 2012);
87 CCS learning from the LNG sector: a report for the Global CCS Institute (Gobal Carbon Capture and Storage (CCS) Institute, 2013); https://hub.globalccsinstitute.com/publications/ccs-learning-lng-sector-report-global-ccs-institute/36-lng-facilities
88 International Energy Agency (IEA). *World Energy Statistics* (2016); https://www.iea.org/statistics/relateddatabases/worldenergystatisticsandbalances/
89 Nawaz, M. & Jobson, M. Synthesis and optimization of demethanizer flowsheets for low temperature separation processes. *Distillation Absorption*, 79-84 (2010).
90 Abrahams, L. S., Samaras, C., Griffin, W. M. & Matthews, H. S. Life cycle greenhouse gas emissions from US liquefied natural gas exports: implications for end uses. *Environmental science & technology* **49**, 3237-3245 (2015).
91 Venkatesh, A., Jaramillo, P., Griffin, W. M. & Matthews, H. S. Uncertainty in life cycle greenhouse gas emissions from United States natural gas end-uses and its effects on policy. *Environmental science & technology* **45**, 8182-8189 (2011).
92 Okamura, T., Furukawa, M. & Ishitani, H. Future forecast for life-cycle greenhouse gas emissions of LNG and city gas 13A. *Applied Energy* **84**, 1136-1149 (2007).
93 Agarwal, R. *et al.* LNG regasification terminals: the role of geography and meteorology on technology choices. *Energies* **10**, 2152 (2017).
94 Upstream Oil & Gas. (Wood Mackenzie, 2019); https://www.woodmac.com/our-expertise/capabilities/upstream-oil-and-gas/
95 Jialiang, L., Suping, Z., Yongxin, H. & Yuping, S. Key issues in the great-leap-forward development of natural gas industry and the exploitation of giant gas fields in China. *Natural Gas Industry* **33**, 13-18 (2013).
96 CNPC. (China National Petroluem Corporation). Management of natural gas development. (Beijing China, 206);
97 Arps, J. J. Analysis of decline curves. *Transactions of the AIME* **160**, 228-247 (1945).
98 Wei, Y. *et al.* Comparative analysis of development characteristics and technologies between shale gas and tight gas in China. *Nat Gas Ind* **6**, 64-68 (2017).
99 ERINDRC. (Energy Research Institute National Development And Reform Commission). Collected Research Works on China’s Energy Issues 2011−2012. (Beijing, China, 2013);

100 Qin, Y., Edwards, R., Tong, F. & Mauzerall, D. L. Can switching from coal to shale gas bring net carbon reductions to China? *Environmental science & technology* **51**, 2554-2562 (2017).
101 Harrison, M. R. *Methane emissions from the natural gas industry*. (US Environmental Protection Agency, National Risk Management Research Laboratory, 1996).
102 Harrison, M. *et al.* Natural Gas Industry Methane Emission Factor Improvement Study Final Report: Cooperative Agreement No. XA-83376101. (2011).
103 Rabchuk, V., Ilkevich, N. & Kononov, Y. A study of methane leakage in the Soviet natural gas supply system. *Sibirian Academy of Science, Irkutsk* (1991).
104 Mitchell, C., Sweet, J. & Jackson, T. A study of leakage from the UK natural gas distribution system. *Energy policy* **18**, 809-818 (1990).
105 Le Fevre, C. Methane Emissions: from blind spot to spotlight. (2017).
106 Balcombe, P. *et al.* The natural gas supply chain: the importance of methane and carbon dioxide emissions. *ACS Sustainable Chemistry & Engineering* **5**, 3-20 (2016).
107 Ishkov, A. *et al.* Understanding methane emissions sources and viable mitigation measures in the natural gas transmission systems: Russian and US experience, in *International Gas Union Research Conference.*
108 Anifowose, B. & Odubela, M. Methane emissions from oil and gas transport facilities–exploring innovative ways to mitigate environmental consequences. *Journal of Cleaner Production* **92**, 121-133 (2015).
109 Lechtenböhmer, S. *et al.* Tapping the leakages: Methane losses, mitigation options and policy issues for Russian long distance gas transmission pipelines. *International journal of greenhouse gas control* **1**, 387-395 (2007).
110 Venugopal, S. The effective management of methane emissions from natural gas pipelines, in *Greenhouse Gas Control Technologies-6th International Conference.* 1293-1298 (Elsevier).
111 Burnham, A. *et al.* Life-cycle greenhouse gas emissions of shale gas, natural gas, coal, and petroleum. *Environmental science & technology* **46**, 619-627 (2011).
112 Rioux, B. *et al.* The economic impact of price controls on China's natural gas supply chain. *Energy Economics* **80**, 394-410 (2019).
113 Ishwaran, M. *et al.* in *China's Gas Development Strategies* 197-232 (Springer, 2017).
114 Trade map: Trade statistics for international business development. (International Trade Center (ITC), 2019); https://www.trademap.org
115 *General Administration of Customs of China* (2019); http://www.customs.gov.cn/
116 Kang, Z. Natural gas supply-demand situation and prospect in China. *Natural Gas Industry B* **1**, 103-112 (2014).
117 Lin, B. & Wang, T. Forecasting natural gas supply in China: production peak and import trends. *Energy Policy* **49**, 225-233 (2012).
118 Chang, Y., Liu, X. & Christie, P. Emerging shale gas revolution in China. *Environmental science & technology* **46**, 12281-12282 (2012).
119 Development Plan of Shale Gas (2016-2020) (National Energy Administration of China, 2016); http://www.gov.cn/xinwen/2016-09/30/content_5114313.htm
120 Policy of shale gas industry (National Energy Administration, 2013); http://zfxxgk.nea.gov.cn/auto86/201310/t20131030_1715.htm
