# Supplementary material for: Carbon footprint of global natural gas supplies to China
Source: Nat Commun. 2020 Feb 11;11:824. doi: 10.1038/s41467-020-14606-4 (PMC7012848; doi:10.1038/s41467-020-14606-4)
Supplement: Supplementary file 3 — Description of Additional Supplementary Files [file 41467_2020_14606_MOESM3_ESM.pdf]

Description of Additional Supplementary Files

File Name: Supplementary Data 1

Description: Share of gas supply from each field, current and projection (%)

File Name: Supplementary Data 2

Description: Field-specific inputs and data sources

File Name: Supplementary Data 3

Description: Transmission distances between gas fields and supply destinations for gas fields conneted to Chinese pipeline system(km)

File Name: Supplementary Data 4

Description: Electricity grid mix of different gas production countries
