# Supplementary material for: Carbon footprint of global natural gas supplies to China
Source: Nat Commun. 2020 Feb 11;11:824. doi: 10.1038/s41467-020-14606-4 (PMC7012848; doi:10.1038/s41467-020-14606-4)
Supplement: Supplementary file 8 — Reporting Summary [file 41467_2020_14606_MOESM8_ESM.pdf]

natureresearch

Corresponding author(s): Yu Gan

Last updated by author(s): Dec 28, 2019

Reporting Summary

Nature Research wishes to improve the reproducibility of the work that we publish. This form provides structure for consistency and transparency in reporting. For further information on Nature Research policies, see Authors & Referees and the Editorial Policy Checklist.

Statistics

For all statistical analyses, confirm that the following items are present in the figure legend, table legend, main text, or Methods section.

n/a	Confirmed	
☒	☐	The exact sample size (*n*) for each experimental group/condition, given as a discrete number and unit of measurement
☒	☐	A statement on whether measurements were taken from distinct samples or whether the same sample was measured repeatedly
☒	☐	The statistical test(s) used AND whether they are one- or two-sided<br>*Only common tests should be described solely by name; describe more complex techniques in the Methods section.*
☒	☐	A description of all covariates tested
☒	☐	A description of any assumptions or corrections, such as tests of normality and adjustment for multiple comparisons
☒	☐	A full description of the statistical parameters including central tendency (e.g. means) or other basic estimates (e.g. regression coefficient) AND variation (e.g. standard deviation) or associated estimates of uncertainty (e.g. confidence intervals)
☒	☐	For null hypothesis testing, the test statistic (e.g. *F*, *t*, *r*) with confidence intervals, effect sizes, degrees of freedom and *P* value noted<br>*Give P values as exact values whenever suitable.*
☒	☐	For Bayesian analysis, information on the choice of priors and Markov chain Monte Carlo settings
☒	☐	For hierarchical and complex designs, identification of the appropriate level for tests and full reporting of outcomes
☒	☐	Estimates of effect sizes (e.g. Cohen's *d*, Pearson's *r*), indicating how they were calculated

Our web collection on statistics for biologists contains articles on many of the points above.

Software and code

Policy information about availability of computer code

Data collection	The present study does not involve the collection of new raw data.
Data analysis	Microsoft Office Professional Plus 2016

For manuscripts utilizing custom algorithms or software that are central to the research but not yet described in published literature, software must be made available to editors/reviewers. We strongly encourage code deposition in a community repository (e.g. GitHub). See the Nature Research guidelines for submitting code & software for further information.

Data

Policy information about availability of data

All manuscripts must include a data availability statement. This statement should provide the following information, where applicable:

- Accession codes, unique identifiers, or web links for publicly available datasets
- A list of figures that have associated raw data
- A description of any restrictions on data availability

Data availability. All data regarding the parameters used in the natural gas LCA model and data sources are documented in Supplementary Note 1. Field-specific parameters input to the LCA model and the corresponding data sources are presented in Supplementary Data 1. The field-specific data were obtained from various sources including statistics reports, industrial technical papers, and research articles. Less than 10% of the inputs were from commercial dataset (https://www.woodmac.com/our-expertise/capabilities/upstream-oil-and-gas/)49 to fill the data gap of publicly available data. All other data used in the study are given in Supplementary Data 2, 3 and 4. The source data underlying all figures in the main manuscript and Supplementary Information are provided as a Source Data file.

Field-specific reporting

Please select the one below that is the best fit for your research. If you are not sure, read the appropriate sections before making your selection.

☐ Life sciences ☐ Behavioural & social sciences ☒ Ecological, evolutionary & environmental sciences

For a reference copy of the document with all sections, see nature.com/documents/nr-reporting-summary-flat.pdf

Ecological, evolutionary & environmental sciences study design

All studies must disclose on these points even when the disclosure is negative.

Study description	The study applied an engineering-based model to calculate the energy consumption and greenhouse gas emissions for natural gas production from different gas fields. The values of parameters in the engineering-based model and gas field-specific parameters were obtained from established modeling tools, public statistics, research articles, scientific/technical and commercial data reports. The present study does not involve the collection of new raw data.
Research sample	The present study does not involve the collection of new raw data and data sampling.
Sampling strategy	The present study does not involve the collection of new raw data and data sampling.
Data collection	The present study does not involve the collection of new raw data.
Timing and spatial scale	The present study does not involve the collection of new raw data.
Data exclusions	The present study does not involve the collection of new raw data and data exclusions.
Reproducibility	The present study does not involve experiments.
Randomization	The present study does not involve allocation of samples and randomization control.
Blinding	The present study does not involve data acquisition and blinding.

Did the study involve field work? ☐ Yes ☒ No

Reporting for specific materials, systems and methods

We require information from authors about some types of materials, experimental systems and methods used in many studies. Here, indicate whether each material, system or method listed is relevant to your study. If you are not sure if a list item applies to your research, read the appropriate section before selecting a response.

Materials & experimental systems

n/a	Involved in the study
☒	☐ Antibodies
☒	☐ Eukaryotic cell lines
☒	☐ Palaeontology
☒	☐ Animals and other organisms
☒	☐ Human research participants
☒	☐ Clinical data

Methods

n/a	Involved in the study
☒	☐ ChIP-seq
☒	☐ Flow cytometry
☒	☐ MRI-based neuroimaging
